# Integrated gene analyses of de novo variants from 46,612 trios with autism and developmental disorders

**DOI:** 10.1073/pnas.2203491119

**Published:** 2022-11-11

**Authors:** Tianyun Wang, Chang N. Kim, Trygve E. Bakken, Madelyn A. Gillentine, Barbara Henning, Yafei Mao, Christian Gilissen, The SPARK Consortium, Tomasz J. Nowakowski, Evan E. Eichler

**Affiliations:** ^a^Department of Genome Sciences, University of Washington School of Medicine, Seattle, WA 98195;; ^b^Department of Medical Genetics, Center for Medical Genetics, Peking University Health Science Center, Beijing, 100191, China;; ^c^Neuroscience Research Institute, Peking University, Key Laboratory for Neuroscience, Ministry of Education of China & National Health Commission of China, Beijing, 100191, China;; ^d^Department of Anatomy, University of California, San Francisco, CA 94143;; ^e^Allen Institute for Brain Science, Seattle, WA 98109;; ^f^Bio-X Institutes, Key Laboratory for the Genetics of Developmental and Neuropsychiatric Disorders, Ministry of Education, Shanghai Jiao Tong University, Shanghai, 200030, China;; ^g^Department of Human Genetics, Radboud Institute for Molecular Life Sciences, Radboud University Medical Center, 6500 HB Nijmegen, The Netherlands;; ^h^The Simons Foundation, New York, NY 10010;; ^i^The Eli and Edythe Broad Center of Regeneration Medicine and Stem Cell Research, University of California, San Francisco, CA 94143;; ^j^Department of Psychiatry and Behavioral Sciences, University of California, San Francisco, CA 94143;; ^k^HHMI, University of Washington, Seattle, WA 98195

**Keywords:** de novo variants, neurodevelopmental disorder, protein–protein interaction, single-nuclei transcriptome

## Abstract

Most genetic studies consider autism spectrum disorder (ASD) and developmental disorder (DD) separately despite overwhelming comorbidity and shared genetic etiology. Here, we analyzed de novo variants (DNVs) from 15,560 ASD (6,557 from SPARK) and 31,052 DD trios independently and also combined as broader neurodevelopmental disorders (NDDs) using three models. We identify 615 NDD candidate genes (false discovery rate [FDR] < 0.05) supported by ≥1 models, including 138 reaching Bonferroni exome-wide significance (*P* < 3.64e–7) in all models. The genes group into five functional networks associating with different brain developmental lineages based on single-cell nuclei transcriptomic data. We find no evidence for ASD-specific genes in contrast to 18 genes significantly enriched for DD. There are 53 genes that show mutational bias, including enrichments for missense (*n* = 41) or truncating (*n* = 12) DNVs. We also find 10 genes with evidence of male- or female-bias enrichment, including 4 X chromosome genes with significant female burden (*DDX3X*, *MECP2*, *WDR45*, and *HDAC8)*. This large-scale integrative analysis identifies candidates and functional subsets of NDD genes.

## Significance

Neurodevelopmental disorders (NDDs) are a group of heterogeneous disorders encompassing both autism spectrum disorder (ASD) and developmental disorder (DD). We searched for genes enriched for de novo variants from 15,560 ASD and 31,052 DD parent–child trios independently and combined as a broader NDD group using three models. We identify 615 candidates (false discovery rate [FDR] < 0.05) supported by one or more model, 138 of which reach exome-wide significance (*P* < 3.64e–7) in all models. NDD genes group into five functional networks with distinct patterns of single-cell expression in the developing brain. We find no ASD-specific genes, although 18 genes are specifically enriched for DD, and we identify 53 genes with mutational bias as well as 10 genes with sex bias. This large-scale integrative analysis provides candidates for future investigation.

Neurodevelopmental disorders (NDDs) are a group of heritable disorders that are twice as likely to affect males than females and whose prevalence continues to increase, in part, due to improved and increased pediatric ascertainment (1). Among NDDs, autism spectrum disorder (ASD), developmental disorder (DD), intellectual disability (ID), and attention-deficit/hyperactivity disorder (ADHD) show considerable heterogeneity, both genetically and clinically, and often present some of the greatest sex disparities in prevalence (2–7). Individuals with different primary NDD diagnoses frequently present overlapping phenotypes. For example, ADHD has been observed as the most common co-occurring disorder among individuals with an ID diagnosis, followed by 15 to 40% of individuals presenting with ASD (8–11). Similarly, ∼30% of individuals with an ASD diagnosis also show some level of cognitive impairment, and ∼30 to 40% of cases co-occur with ADHD (5, 12). This significant diagnostic overlap among NDD groups has long suggested a shared genetic etiology, at least among a subset of cases (13).

De novo variants (DNVs) that disrupt or alter protein-coding gene function significantly contribute to NDDs. This genetic signal has facilitated the discovery of hundreds of risk genes over the last decade (14–16). Most studies to date have focused on a single phenotypic group (e.g., either on ASD (17) or DD (18)) in an effort to identify genes that specifically contribute to that phenotype. The locus heterogeneity of these disorders, however, has limited power to identify genes with rare DNVs given that more than half of the genes still await a sufficient number of cases to reach statistical significance (19). Moreover, different studies have tended to apply their preferred statistical or evolutionary models to identify genes with an excess of DNVs. This has led to the emergence of slightly different gene sets from the same parent–child trio data (17, 19).

**Fig. 1.**
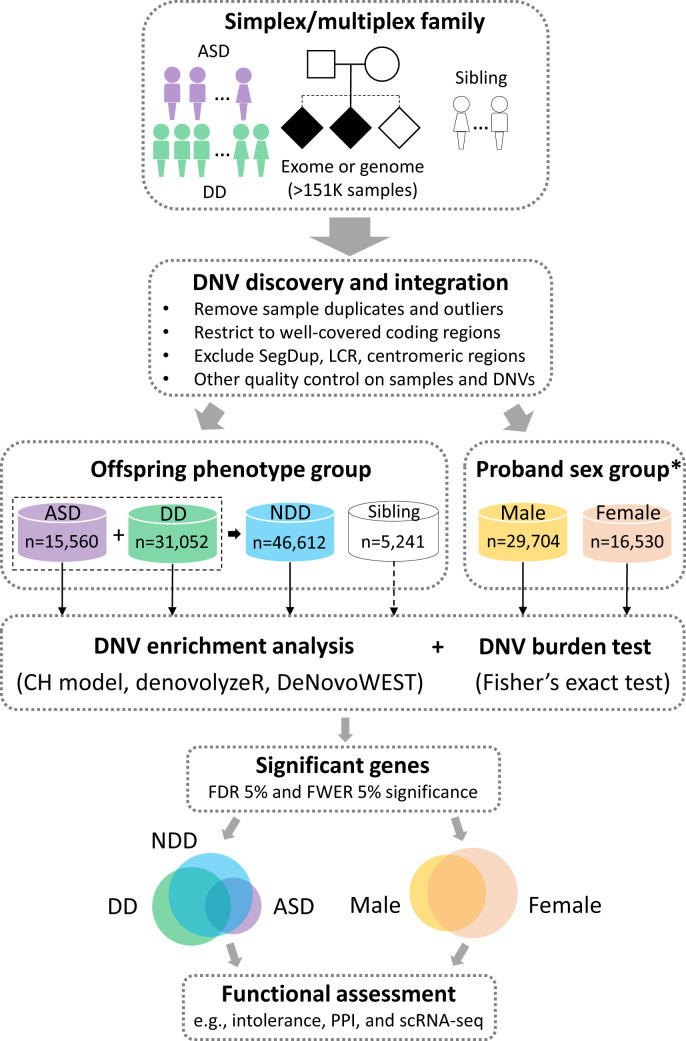
Study workflow. DNVs from >151,000 samples, including both simplex and multiplex families with a primary diagnosis of ASD or DD, were integrated with strict QC and filtering measures applied (*SI Appendix*, *Supplementary Methods*). De novo enrichment analysis was performed independently in ASD (*n* = 15,560), DD (*n* = 31,052), and NDD (*n* = 46,612) groups, and probands with sex information available were grouped by males and females, in parallel using three statistical models (CH model, denovolyzeR, and DeNovoWEST). Siblings were also analyzed using the CH model and denovolyzeR, but not run for DeNovoWEST due to the small sample size (*n* = 5,241). Significant genes were used for downstream analyses for the identification of risk genes and the comparison between phenotype and sex. *Sex information is available for the majority (99.2%, 46,234/46,612) of the probands. SegDup: segmental duplications; LCR: low-complexity regions.

In this study, we integrate DNVs from 11 cohorts with a primary diagnosis of ASD or DD from 46,612 parent–child trios, 6,557 ASD trios of which are from Simons Foundation Powering Autism Research for Knowledge (SPARK, https://sparkforautism.org), and apply three different statistical models (chimpanzee–human divergence [CH] model (19, 20), denovolyzeR (21), and DeNovoWEST (18)) (Fig. 1). Briefly, the CH model estimates the number of expected DNVs by incorporating locus-specific transition, transversion, and indel rates, the gene length, and null expectation based on chimpanzee–human coding sequence divergence; denovolyzeR estimates mutation rates, considers the triplet context, and adjusts divergence based on macaque–human gene comparisons; DeNovoWEST applies the same underlying mutation rate as denovolyzeR but also incorporates gene-based weighting with missense clustering to provide the most sensitive set of candidate genes. In addition to applying standard statistical thresholds of significance, we prioritized genes as higher confidence if they were predicted by more than one or all of these three models and flag potential artifacts in previous call sets based on exclusivity of variant calls to a particular study.

The goal of this analysis is fourfold: (1) discover candidate genes by combining ASD- and DD-diagnosed individuals as a broader NDD cohort; (2) distinguish ASD- from DD-enriched genes and test whether any genes show an enrichment in one diagnosis over the other (22); (3) ascertain genes that favor a particular class of variant (i.e., missense over likely gene-disruptive [LGD]); and (4) identify genes that are biased for DNVs either among females or males. We further explore the protein–protein interaction (PPI) networks and expression properties of these genes both in bulk tissue and single-cell RNA sequencing (scRNA-seq) data to reveal properties regarding the tissues and functional neural networks affected by the disruption of these genes.

## Results

### DNV Discovery and Integration across Cohorts.

We integrated exome and genome sequencing data from over 44,800 parent–child families with more than 151,000 individuals from 11 cohorts (17, 18, 23–28), in which children received a primary diagnosis of ASD or DD (*SI Appendix*, Table S1). We split the cohorts into two subsets based on whether the underlying Illumina sequencing data were available for reanalysis and variant recalling. We identified DNVs by reanalyzing underlying sequence data using FreeBayes and GATK as described previously (29) for five cohorts (recalled subset: 46.4% of families with 60,868 exomes and 9,304 genomes) and retrieved published DNVs from the other six cohorts (no-recall subset: 53.6% of families with 81,052 exomes) (*SI Appendix*, Table S2). After DNV discovery and integration, we further performed stringent quality control (QC) and filtering on both the recalled and no-recall subsets to better harmonize the DNVs (*Materials and Methods*).

After QC on both samples and variants, the harmonized DNV set came from 15,560 ASD (6,557 from SPARK) and 31,052 DD patients as well as 5,241 unaffected siblings (3,034 from SPARK). This set includes 6,921 de novo LGD (dnLGD, including frameshift, stop-gain, splice-donor, or splice-acceptor) variants, 32,774 de novo missense (dnMIS) variants, as well as 11,706 de novo synonymous (dnSYN) variants. Among the dnMIS variants, we further classify 5,946 as severe based on a combined annotation dependent depletion (CADD) score (30, 31) greater than 30 (dnMIS30, version 1.3 on hg19), which rank among the top 0.1% of variants with the most severe predicted effect (Dataset S1). There are 11,409 genes with nonsynonymous DNVs in probands versus 2,742 genes in siblings, with 37.7% (4,304/11,409) of the genes in probands and 83.7% (2,296/2,742) of genes in siblings with only one DNV (*SI Appendix*, Fig. S1). Comparing the overall mutation rate for the recalled and no-recall sample sets gave similar DNV frequency (∼0.78 nonsynonymous DNVs per proband). When considering cohorts by phenotype, we find that DD probands (1.04 DNV per person) show the highest DNV rate followed by ASD probands (0.93 DNV per person) and then siblings (0.88 DNV per person) (*SI Appendix*, Fig. S2 and Table S2), although the platform and methodological differences among each cohort may partly contribute to the discrepancy on DNV rate.

### DNV-Enriched NDD Candidate Genes.

To identify genes with a significant excess of DNVs, we applied three statistical models (CH model (19, 20), denovolyzeR (21), and DeNovoWEST (18)) to the ASD and DD cohorts based on their primary diagnoses independently and then combined as one broader NDD group (Fig. 1). After excluding genes that showed evidence of DNV significance among unaffected siblings (*n* = 7; Dataset S2), we identify 615 candidate genes in the combined NDD group with an excess of DNVs by one or more of the three models (union false discovery rate [FDR] <0.05, DNV count >2), 59.3% (365/615) genes of which are intolerant (probability of being loss-of-function intolerant [pLI] >0.9) and show expression in Genotype-Tissue Expression (GTEx) brain tissues. Of note, we consider these 615 genes to be the most sensitive set with the lowest confidence (LC615 genes). This set should be regarded as candidates for future investigation. Among the LC615 genes, we defined 237 genes with moderate confidence (MC237 genes) that reach <5% FDR by all three models (intersection FDR <0.05, DNV count >2); 80.2% (190/237) of MC237 genes are intolerant (pLI >0.9) and show expression in GTEx brain tissues. We further defined the highest confidence set of 138 genes (HC138 genes) in which the excess of DNVs meets exome-wide significance supported by all three models (intersection family-wise error rate [FWER] 5%, *P* < 3.64e–7, DNV count > 2) (Table 1 and Dataset S3 and *SI Appendix*, Fig. S3), and 86.2% (119/138) of HC138 genes are intolerant (pLI > 0.9) and show expression in GTEx brain tissues. Across the three models, DeNovoWEST shows the greatest sensitivity because it incorporates missense clustering in addition to strict counts of DNVs (18). We observe similar trends in our independent analyses of the ASD and DD cohorts (Table 1 and *SI Appendix*, Fig. S4 and Datasets S4 and S5). Candidate genes, as expected, are significantly more likely to be intolerant to variation (*SI Appendix*, Fig. S5).

**Table 1.** Genes with significant excess of DNVs across phenotype and sex groups

Group	Samples	No. DNVs			FDR 5% significant genes (q < 0.05)					FWER 5% significant genes (*P* < 3.64e–7)				
		dnLGD	dnMIS	dnMIS30	CH model	denovolyzeR	DeNovoWEST	Union	Intersection	CH model	denovolyzeR	DeNovoWEST	Union	Intersection
Offspring phenotype group														
ASD	15,560	1,661	9,187	1,454	70	52	113	133	41	26	22	27	39	16
DD	31,052	4,931	20,588	4,084	352	293	414	511	233	181	166	196	241	136
NDD*	46,612	6,592	29,775	5,538	399	323	479	615 (LC615)	237 (MC237)	208	171	190	264	138 (HC138)
Sibling	5,241	329	2,999	408	3	6	—	7	—	1	1	—	2	—
Proband sex group														
Male	29,704	3,820	18,590	3,343	233	191	308	385	141	117	105	104	149	77
Female	16,530	2,716	10,986	2,149	222	193	208	277	152	117	112	109	144	85

Two levels of significance, FDR 5% and FWER 5%, were assessed based on the union and intersection of three statistical models. The lower confidence set (LC615) is based on the union of all genes at FDR 5% significance observed in any one or more of the three models; the MC237 set is based on the intersection of FDR 5% supported by all three models; the highest confidence set (HC138) is based on the intersection of genes with FWER 5% significance supported by all three models. The FDR 5% significance threshold was corrected by the Benjamini-Hochberg method for all of the genes in each model (18,946 genes in CH model, 19,618 genes in denovolyzeR, and 18,762 genes in DeNovoWEST); the FWER 5% significance threshold was corrected by the Bonferroni method for 19,618 genes (the largest among three models) and seven tests in the analyses (tests for dnLGD, dnMIS, and dnMIS30 variants in CH model; dnLGD and dnMIS variants in denovolyzeR; and DNVs and dnMIS variants in DeNovoWEST). Genes with DNV significance in siblings were excluded from all sets (*n* = 7).

^*^NDD = ASD + DD. Details regarding the NDD risk genes can be found in Datasets S2–S5, S9, and S10.

Among the MC237 genes, 30 show increased statistical significance when compared to three previous large-scale reports (17–19) (*SI Appendix*, Fig. S6 and Dataset S6). Among the 30 candidates with significance at 5% FDR based on all three models, we identified 21 genes (*MED13*, *NALCN*, *NF1*, *ASXL2*, *SPTBN1*, *CTNND1*, *TSC1*, *GGNBP2*, *AAAS*, *PSMD12*, *RORA*, *CYFIP2*, *PPP3CA*, *PSPH*, *PLCG2*, *ATP1A2*, *POLR3B*, *KCNT2*, *HIST1H4J*, *CPA6*, and *RALA*) with additional evidence of pathogenicity (including case reports) within the Development Disorder Genotype-Phenotype Database (DDG2P), SFARI Gene, Online Mendelian Inheritance in Man (OMIM) database, and PubMed. Further restricting to genes intolerant to mutation (pLI > 0.9) and with medium to high expression in the brain (GTEx transcripts per million [TPM] > 10) identifies three potential associations (*PABPC1*, *MARK2*, and *GSK3B*) (Dataset S6). We identify or confirm three genes (*MED13*, *NALCN*, and *PABPC1*) among the most stringent set of HC138 genes as now reaching exome-wide significance in this large dataset, further confirming the association of *MED13* and *NALCN* with NDDs based on recent clinical reports (32, 33) (*SI Appendix*, Fig. S6). We also note that there are 25 genes with potential associations with NDDs if we consider exome-wide significance more broadly by the union of any one or more of the models instead of requiring all three models to intersect (Dataset S6). These genes, thus, represent an important resource for future clinical and basic research investigations related to NDDs.

In an effort to provide additional support for the role of these NDD candidate genes, we performed a case-control mutational burden for ultra-rare gene-disruptive mutations in a recently released cohort of 20,817 autism families (32,783 individuals) from SPARK (34), which had not been included as part of our initial meta-analysis. Specifically, we compared the frequency of rare LGD (minor allele frequency [MAF] <0.01%) mutations among the LC615 genes in this autism cohort (*n* = 21,200 cases) with the Exome Aggregation Consortium (ExAC) nonpsychiatric subset (*n* = 45,376 controls), carefully controlling for platform and sequence-read-depth differences (*Materials and Methods*). In total, this replication identified 52 genes in which additional evidence of case-control mutational burden significance was identified (FDR < 0.05, one-sided Fisher’s exact test). This included 25 genes in the HC138 set, 11 genes among MC237 set, and 16 genes restricted to the LC615 set (Dataset S6). After literature and database searches (e.g., DDG2P, SFARI Gene, OMIM, PubMed), filtering for GTEx brain tissue expression, and pLI intolerance as mentioned above, we highlight five genes (*PABPC1*, *MARK2*, *SF3B2*, *CDKN2AIP*, and *RTF1*) as compelling candidates with a potential association with NDDs and are replicated by a case-control design with autism probands.

### Diagnostic Specificity of DNV-Enriched Genes.

**Fig. 2.**
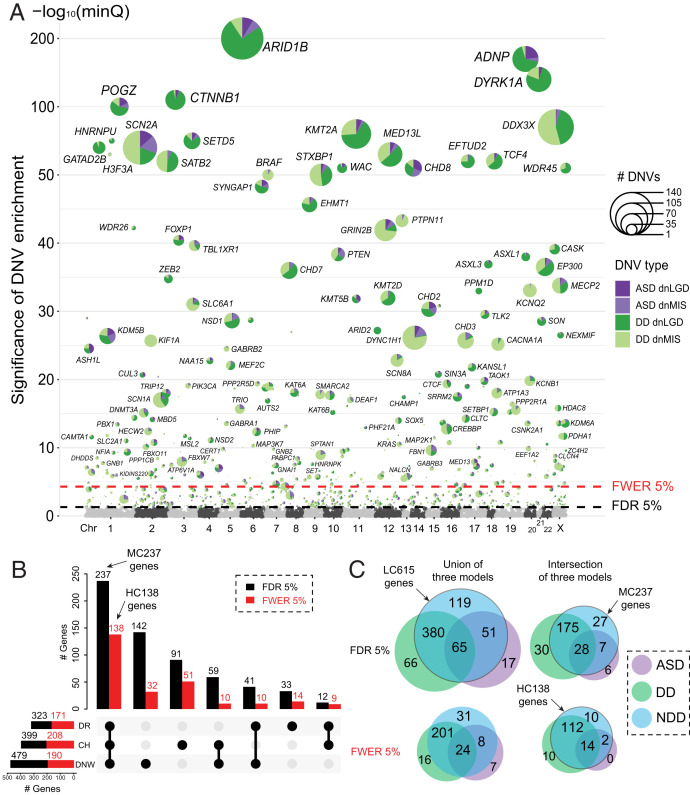
De novo enrichment analysis and significant genes by model and phenotype. (*A*) The smallest q value (minQ) after Benjamini-Hochberg correction of each gene across the three models was plotted in alphabetic order of gene name by chromosome. The LC615 genes reaching union FDR 5% significance were plotted with the number of dnLGD and dnMIS variants in the ASD and DD cohorts scaled in pie charts; the HC138 genes reaching the intersection FWER 5% significance were additionally labeled with gene name. (*B*) The number of genes reaching FDR 5% (black bar) and FWER 5% (red bar) significance identified by each of the three models (DR: denovolyzeR; CH: CH model; DNW: DeNovoWEST) in the combined NDD set. (*C*) Cohort overlap among low-confidence (*n* = 615, FDR 5%) and high-confidence (*n* = 138 FWER 5%) gene sets considering the ASD and DD cohorts separately and as one group (NDD). Genes were compared based on the union of three models for DNV enrichment versus only those that were observed by all three (intersection).

We considered cohorts with a primary diagnosis of ASD and DD separately using the same criteria and then compared the groups to identify genes potentially unique to a specific diagnosis. This analysis identifies several genes enriched for DNVs that are unique to each primary diagnosis or combined (intersection FDR 5%): ASD (*n* = 6), DD (*n* = 30), and NDD (n = 27) (Fig. 2). Among the five genes with unique intersection FDR significance in ASD, three (*TBR1*, *MPHOSPH10*, and *PAPOLG*) also show evidence of DNVs in DD patients, which suggests that the screening of further DD samples will likely yield additional cases and eliminate the ASD specificity. Although two genes (*PAX5* and *FXYD5*) currently show no DNVs among DD patients and thus appear ASD specific, none reach exome-wide significance. We suggest that these should be regarded as either unlikely or low-confidence “ASD-specific” genes. In contrast to ASD, there is compelling evidence for DD-specific genes that are not associated or rarely associated with ASD. Among the 30 genes that reach FDR significance (intersection of models) only in DD but neither ASD nor the combined NDD set, there are 21 genes where no DNV has yet been observed among ASD individuals (Fig. 2). As expected, genes reaching significance in the broader NDD supergroup show DNVs in both ASD and DD patients, but 10 genes reach genome-wide significance only when cohorts are combined as a broader NDD group (*MYT1L*, *PHF21A*, *FBN1*, *PBX1*, *GNB2*, *PABPC1*, *CLCN4*, *NALCN*, *PSMC5*, and *CERT1*), highlighting the value of this integrated analysis. In addition, we also repeated de novo enrichment analyses using the same three models (CH model, denovolyzeR, and DeNovoWEST) with a more balanced sample size. Two approaches were taken: (1) we downsampled DD to match ASD cohort size, and (2) we increased ASD to match DD sample size (*SI Appendix*, *Supplementary Analyses*). Both analyses with matched sample sizes for ASD and DD cohorts showed that sample size alone unlikely underlies the paucity of true ASD-specific genes at least for a de novo mutation model.

**Fig. 3.**
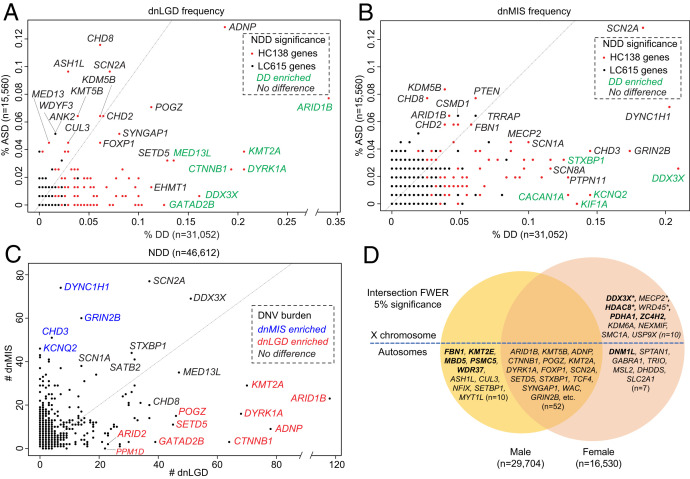
Genes with phenotype, variant class, and sex-biased DNV burden. (*A*) dnLGD and (*B*) dnMIS variant frequencies in ASD (y axis) and DD (x axis) patients were plotted for all LC615 genes in the combined NDD group, with the HC138 genes in red and others in black dots. Genes with enriched DNVs in DD over ASD patients were in green. (*C*) Number of dnLGD and dnMIS variants for all genes with DNVs in the combined NDD group. Example genes with significant burden of dnLGD (red) or dnMIS (blue) variants compared to the other variant class are labeled with gene name in color. (*D*) Genes with potential sex bias. Males and females were treated as two separate groups and genes that reached intersection FWER significance (*P* < 3.64e–7, by all three models) for DNVs in males (*Left*) and females (*Right*) as opposed to both sexes (center of Venn diagram). The genes in bold are those with sex-specific significance and without any significance observed in the other sex group. Genes with asterisks are high-confidence candidates with sex-biased DNV enrichment.

**Fig. 4.**
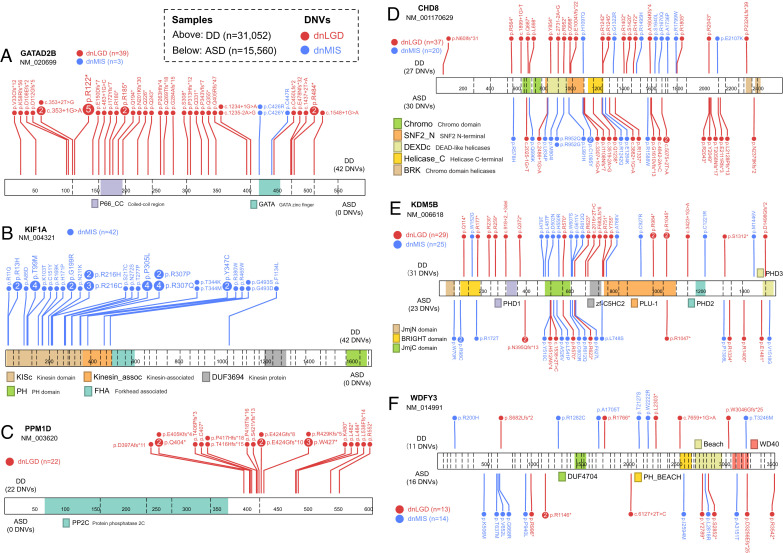
Example genes with variant class and phenotype-specific DNV pattern. Linear protein diagrams are present with size and exons split by vertical dashed lines. Domains are indicated in color blocks with a short description; the total number of dnLGD (red) and dnMIS (blue) variants for each gene was also provided. Recurrent DNVs are indicated by larger circles with the number of recurrences inside. Number of samples plotted: ASD (*n* = 15,560) and DD (*n* = 31,052). (*A*) GATAD2B with DNVs exclusively in DD patients and enriched for dnLGD variants. (*B*) KIF1A only has dnMIS variants and are exclusively in DD patients. (*C*) PPM1D only has dnLGD variants and are exclusively in DD patients. (*D*) CHD8, (*E*) KDM5B, and (*F*) WDYF3 have dnLGD and dnMIS variants in both DD and ASD patients, although no phenotype-specific significance, but tend to have more DNVs in ASD than in DD patients when considering the sample size.

Comparing the relative frequency of DNVs among DD and ASD cohorts identifies genes with a potential bias toward DD or ASD diagnoses, especially when DNVs are further categorized by dnMIS or dnLGD variant class (Fig. 3 and *SI Appendix*, Table S3 and Dataset S7). There are chromatin modifiers among the genes trending toward ASD diagnosis: *CHD8*, *KDM5B*, *ASH1L*, and *KMT5B*—although none of these genes reach significance for ASD enrichment. In contrast to DD patients, a comparison of DNV counts identifies 18 genes with higher DNV burden in DD over ASD cohorts (two-sided Fisher’s exact test, FDR 5% corrected for 20,000 genes) (Table 2). *GATAD2B* and *KIF1A* are exclusive to DD without DNVs in ASD individuals in this study (Fig. 4). Interestingly, *GATAD2B* shows an enrichment only for dnLGD variants, while in contrast, *KIF1A* shows a specific enrichment for dnMIS variants (Fig. 4). Patients with variants in these genes have been described as exhibiting DD and moderate to severe ID (35, 36). Although no gene shows significant ASD-specific burden, 41 of the LC615 genes present with a higher DNV frequency in ASD when compared to DD with sample size adjusted (Fig. 4 and Dataset S7). For example, *CHD8* is highly intolerant to LGD variants (pLI score = 1, loss-of-function observed/expected upper bound fraction [LOEUF] score = 0.082) and shows a 2.22-fold enrichment of DNVs in ASD patients (*n* = 15,560, 18 dnLGD and 12 dnMIS variants) when compared to DD patients (*n* = 31,052, 19 dnLGD and 8 dnMIS variants). Other genes, such as *KDM5B* (1.48-fold) and *WDFY3* (2.90-fold), also have more DNVs in ASD versus DD patients relative to the corresponding sample size.

**Table 2.** Genes with excess DNV burden in DD versus ASD patients

Gene	DD DNV (dnLGD/dnMIS)	ASD DNV (dnLGD/dnMIS)	*P*	q	OR (95% CI)	Significant variant
*ARID1B*	119 (106/13)	22 (12/10)	2.51e–6	5.02e–3	2.7 (1.7–4.5)	DNV&dnLGD
*DDX3X*	115 (50/65)	5 (1/4)	5.41e–15	1.08e–10	11.6 (4.8–36.3)	DNV&dnLGD&dnMIS
*KMT2A*	90 (64/26)	9 (6/3)	2.25e–8	1.50e–4	5 (2.5–11.3)	DNV&dnLGD
*DYRK1A*	80 (64/16)	4 (4/0)	3.11e–10	3.11e–6	10 (3.8–37.8)	DNV&dnLGD
*MED13L*	72 (42/30)	10 (5/5)	1.71e–5	2.14e–2	3.6 (1.9–7.9)	DNV
*SATB2*	68 (32/36)	5 (0/5)	1.31e–7	4.37e–4	6.8 (2.8–21.7)	DNV&dnLGD
*STXBP1*	67 (28/39)	8 (3/5)	7.10e–6	1.18e–2	4.2 (1–10.1)	DNV
*CTNNB1*	62 (60/2)	5 (4/1)	7.84e–7	1.74e–3	6.2 (2.5–19.8)	DNV&dnLGD
*TCF4*	51 (31/20)	4 (3/1)	9.21e–6	1.42e–2	6.4 (2.4–24.4)	DNV
*KMT2D*	47 (30/17)	3 (0/3)	6.63e–6	1.18e–2	7.9 (2.5–39.5)	DNV&dnLGD
*KCNQ2*	45 (0/45)	1 (0/1)	3.01e–7	8.61e–4	22.6 (3.9–907.9)	DNV&dnMIS
*CACNA1A*	43 (3/40)	1 (0/1)	5.05e–7	1.26e–3	21.6 (3.7–868.6)	DNV&dnMIS
*EFTUD2*	43 (31/12)	3 (1/2)	2.67e–5	3.14e–2	7.2 (2.3–36.2)	DNV
*WDR45*	35 (24/11)	1 (1/0)	1.06e–5	1.51e–2	17.6 (3–710.9)	DNV
*CASK*	34 (24/10)	1 (1/0)	1.71e–5	2.14e–2	17.1 (2.9–691.2)	DNV
*GATAD2B*	42 (39/3)	0 (0/0)	5.52e–8	2.21e–4	Inf (5.5–Inf)	DNV&dnLGD
*KIF1A*	42 (0/42)	0 (0/0)	5.52e–8	2.21e–4	Inf (5.5–Inf)	DNV&dnMIS
*CHD7*	51 (31/20)	6 (0/6)	7.36e–6	1.64e–2	Inf (4–Inf)	dnLGD only

Two-sided Fisher’s exact test for DNV counts was performed comparing ASD (*n* = 15,560) and DD (*n* = 31,052) patients. This table shows the 18 genes with significant DNV burden in DD compared to ASD patients, while no gene with significant burden is identified in ASD patients. The significance threshold (FDR 5%) was corrected for 20,000 genes (Benjamini-Hochberg). OR: odds ratio; CI: confidence interval; Inf: infinity.

### Genes with Biased Burden by Variant Class.

In the combined NDD group (*n* = 46,612), most genes (92.7%, 10,578/11,409) show a greater number of dnMIS variants when compared to dnLGD variants, which is consistent with expectations. However, a subset of 7.3% (831/11,409) show the reverse pattern with more dnLGD than dnMIS variants (e.g., *ARID1B*, *ADNP*, *KMT2A*, *DYRK1A*, *CTNNB1*, *MED13L*, *POGZ*, *SETD5*, *GATAD2B*), suggesting different mechanisms of pathogenicity or phenotypic manifestation by mutational class. A direct comparison of dnLGD and dnMIS variant counts for each gene in the combined NDD cohort (*n* = 11,409 genes) identifies 12 genes (*ARID1B*, *ADNP*, *KMT2A*, *DYRK1A*, *CTNNB1*, *SETD5*, *GATAD2B*, *WAC*, *ASXL1*, *ASXL3*, *ARID2*, and *PPM1D*) significantly enriched for dnLGD when compared to dnMIS variants (two-sided Fisher’s exact test, FDR 5%). By contrast, there are 41 genes with significantly greater burden of dnMIS over dnLGD variants (e.g., *DYNC1H1*, *GRIN2B*, *CHD3*, *CACNA1A*, *SCN8A*) (Fig. 3 and Dataset S8). Among the above genes with a bias by variant class, *PPM1D* shows exclusively dnLGD variants (*n* = 22), while 23 genes show only dnMIS variants (e.g., *KIF1A*, *KCNQ2*, *PTPN11*, *SMARCA2*) in this dataset (Fig. 4).

### Sex-Biased DNV Enrichment and Burden Analysis.

**Fig. 5.**
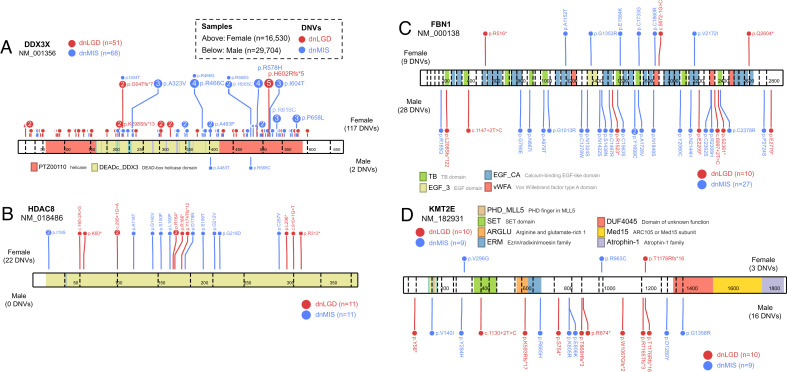
Example genes with sex-biased DNV pattern. Linear protein diagrams are plotted in same way as in Fig. 4. Number of samples plotted: female (*n* = 16,530) and male (*n* = 29,704). (*A*) DDX3X and (*B*) HDAC8 DNVs are almost exclusively in females and with female-only DNV burden and enrichment significance. (*C*) FBN1 and (*D*) KMT2E, while having no sex-biased DNV burden significance, show male-specific DNV enrichment significance and more DNVs in males than females.

The male-biased predominance is well established for NDDs, especially in ASD, but the underlying mechanism remains largely unknown. We further investigated this sex bias in 29,704 male and 16,530 female probands in whom sex information is available (Table 1). Using the three models and treating males and females as two distinct groups, we identified 33 and 28 candidate genes with potential male or female bias for DNVs, respectively, that have no significance in the other sex group (intersection FDR 5%) (Datasets S9 and S10). After applying the most stringent exome-wide significance criteria (intersection FWER 5%), five male-bias (*FBN1*, *KMT2E*, *MBD5*, *PSMC5*, and *WDR37*) and five female-bias (*DDX3X*, *HDAC8*, *PDHA1*, *ZC4H2*, and *DNM1L*) enriched genes were identified (Fig. 3*D*). By directly comparing the DNV counts in males versus females and controlling for sex chromosomes, four genes (*DDX3X*, *MECP2*, *WDR45*, and *HDAC8*) show a significant excess of DNVs in females (two-sided Fisher’s exact test, FDR 5% corrected for 20,000 genes in human genome) (Table 3 and Fig. 5). Notably, all four genes map to the X chromosome with either no DNVs or a low number (≤7) of DNVs observed in males, suggesting that such variants may be lethal during male embryonic development. Although no individual gene shows a significant male excess when comparing DNV counts between the sexes, several genes show interesting trends. For example, *KMT2E* encodes a member of the lysine *N*-methyltransferase-2 family of chromatin modifiers, is associated with O’Donnell-Luria-Rodan syndrome, and shows a male bias in our data (16 DNVs in males versus three in females); finally, *KDM6B* shows 12 DNVs in males compared to two events in females and encodes a histone H3K27 demethylase that specifically demethylates di- or trimethylated lysine-27 (K27) of histone H3 (Table 3 and Fig. 5).

**Table 3.** Top genes with sex-specific DNV enrichment

Gene	Chr	Female DNVs (dnLGD/dnMIS)	Male DNVs (dnLGD/dnMIS)	Minimum *P*	Minimum q	Intersection FWER 5% significance
*DDX3X*	X	117 (51/66)	2 (0/2)	1.95e–120	3.83e–116	Female only
*HDAC8*	X	22 (11/11)	0 (0/0)	5.42e–24	4.11e–21	Female only
*PDHA1*	X	19 (9/10)	2 (0/2)	1.09e–17	7.37e–15	Female only
*ZC4H2*	X	7 (7/0)	1 (0/1)	5.06e–15	2.55e–12	Female only
*DNM1L*	12	11 (1/10)	1 (0/1)	3.18e–11	1.64e–8	Female only
*FBN1*	15	9 (3/6)	28 (7/21)	1.22e–11	3.71e–9	Male only
*KMT2E*	7	3 (1/2)	16 (9/7)	1.79e–11	5.24e–9	Male only
*MBD5*	2	3 (3/0)	15 (11/4)	5.81e–15	3.26e–12	Male only
*PSMC5*	17	1 (0/1)	10 (0/10)	7.61e–12	2.38e–9	Male only
*WDR37*	10	3 (0/3)	10 (0/10)	3.09e–10	1.77e–7	Male only

Independent DNV enrichment analysis was performed in female (*n* = 16,530) and male (*n* = 29,704) patients. This table shows the top 10 genes with evidence of sex-specific DNV enrichment in females and males. The significance thresholds (FDR 5% and FWER 5%) were corrected for multiple testing as in Table 1 males (FDR 5%, corrected for 20,000 genes using Benjamini-Hochberg method, two-sided Fisher’s exact test). Chr: chromosome.

### Functional and Neuronal Properties of NDD Genes.

**Fig. 6.**
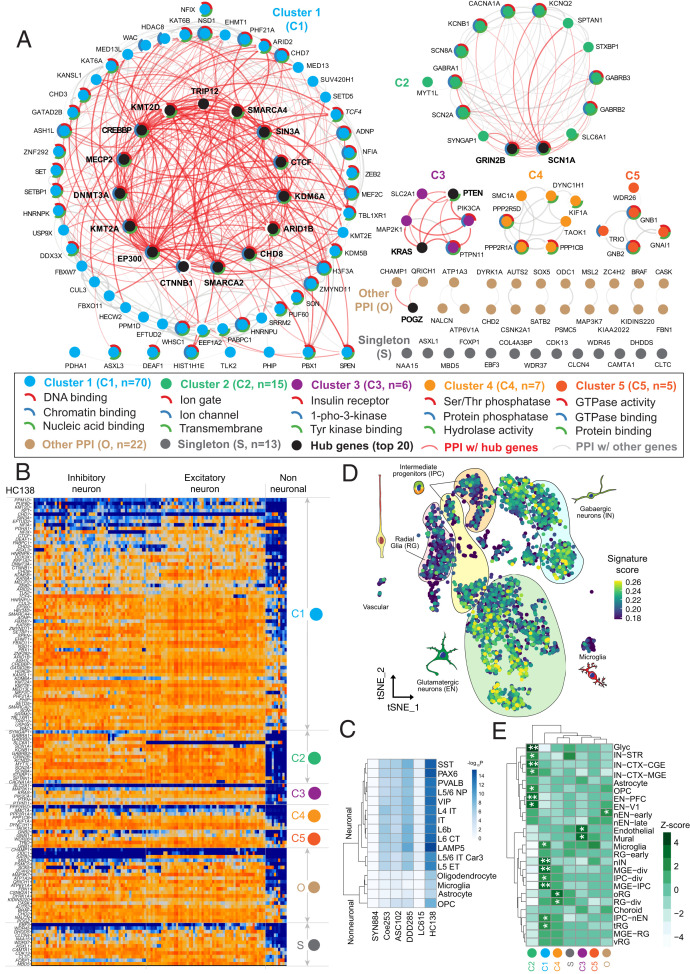
PPI analysis and panneuronal expression of the highest confidence genes. (*A*) PPI analysis identified five main clusters (C1 to C5), as well as 22 genes in another smaller PPI group (O) and 13 singleton genes (S) using STRING for the HC138 genes with the highest confidence. The top three GO functions were indicated by the pie chart in color (top 1 in red, top 2 in blue, and top 3 in green), if applicable, outside each gene dot with a short name in the legend. We also defined 20 top hub genes (black dot and bolded name) supported by at least half of 12 statistical methods in cytoHubba (37). Red arcs designate PPI with hub genes; gray arcs indicate PPI only between non-hub genes. The degree of the color and the width of the arc indicate the degree of the interaction. (*B*) Expression heatmap of the HC138 genes in 120 cell types identified across 6 human neocortical areas grouped by PPI clusters with higher expression (orange) and lower expression (blue). The cell types were grouped by transcriptomic similarity, and the major branches correspond to inhibitory and excitatory neurons and nonneuronal cells. Labels on the *Right Side* indicate clusters based on PPI analysis, and gene names are on the *Left Side*. (*C*) Heatmap of −log_10_-transformed *P* values (Bonferroni corrected for multiple testing) of a Kolmogorov-Smirnov test for the difference in the expression levels of each gene set (rows) in each cell subtype (columns, full name described in *Materials and Methods*) compared to a control set of genes (HC138 and LC615 are the gene sets with the HC and LC identified in this study; DDD285 (18), ASC102 (17), and Coe253 (19) are significant genes reported previously; SYN884, the control set, includes 844 genes with dnSYN variants [*n* > 2] in the 46,612 NDD samples in this study). (*D*) Gene signature score of the HC138 genes computed per cell. IN: interneurons; EN: excitatory neurons; IPC: intermediate progenitor cells; MGE: medial ganglionic eminence; CGE: caudal ganglionic eminence; OPC: oligodendrocyte progenitor cells; tRG: truncated radial glia; oRG: outer radial glia; vRG: ventral radial glia; CTX: cortex; V1: visual cortex; PFC: prefrontal cortex; STR: striatum. (*E*) The heatmap shows the SD from the mean expression value of each cluster of genes. Positive values are up-regulated compared to the mean, and negative values are down-regulated compared to the mean. Significance is derived from bootstrapping and labeled with asterisk (*P < 0.05, **FDR *P* < 0.05 after Benjamini-Hochberg correction). The HC138 genes were used as the background gene set.

We next focused on investigating the functional properties of the highest confidence gene set (HC138) by considering both PPI and gene-expression trends and differences in the adult and developing human brain. A PPI network analysis using STRING-db highlights five distinct network clusters (*P* < 1e–16) (Fig. 6). These include previously described networks such as chromatin binding (Gene Ontology [GO]: 0003682, *P* = 2.25e–22), ion channels (GO: 0022839, *P* = 8.43e–13), lysine degradation (hsa00310, Kyoto Encyclopedia of Genes and Genomes [KEGG], *P* = 6.86e–9), and the GABAergic synapse (hsa04727, KEGG, *P* = 8.78e–8). We identify 20 top “hub” genes, where we observe an excess of PPI interactions supported by at least half of the 12 models in cytoHubba (37) (Dataset S11). Tissue-specific expression analysis (TSEA), as expected, shows enrichment in the brain (*P* = 0.003), with cell-type-specific expression analysis (CSEA) further highlighting expression in specific brain regions from early to late-mid-fetal developmental stages: early-mid- (FDR *P* = 0.002) and late-mid-fetal (FDR *P* = 0.035) amygdala, early fetal cerebellum (FDR *P* = 0.006), early to late fetal cortex (FDR *P* = 1.46e–4), early to early-mid-fetal striatum (FDR *P* = 0.003), and early fetal thalamus (FDR *P* = 0.015). Overall, the HC138 genes are more highly expressed prenatally, consistent with the early developmental impact of variation in these genes and NDDs (*SI Appendix*, Fig. S7).

We also explored the enrichment of NDD candidate genes (ASC102 (17), Coe253 (19), DDD285 (18), and LC615 and HC138 in this study) at cellular resolution from expression datasets obtained from the developing and adult human cerebral cortex (38). Overall, the NDD candidate genes show broad expression across adult human cortical cell types, including inhibitory and excitatory neurons and, to a lesser extent, nonneuronal cells (Fig. 6). All PPI-defined clusters show a similar pattern of panneuronal enrichment. While the signal is less pronounced for the LC615 genes, it is still qualitatively more than the 447 control genes that have more than one nonsynonymous DNV in siblings (*SI Appendix*, Fig. S8). We tested for expression enrichment in neuronal and nonneuronal cells for NDD versus control gene sets by comparing expression levels for cell subtypes (Fig. 6 and *SI Appendix*, Fig. S8) and the number of clusters that express genes for broad cell classes (*SI Appendix*, Figs. S9 and S10). All NDD candidate gene sets are significantly enriched in neuronal subtypes, and all gene sets (except the LC615 genes) are enriched in nonneuronal types (Bonferroni-corrected *P* < 0.05, Kolmogorov-Smirnov test). While different neuronal subtypes show similar enrichments, we note that oligodendrocyte progenitor cells show the greatest enrichment when compared to other nonneuronal cells (Fig. 6). If we use the panneuronal expression signature as a further classifier of functional enrichment, we find that 194 of the LC615 gene set meet these criteria and are panneuronal genes (Dataset S6).

To further dissect the neuronal signature, we investigated single-nucleus transcriptomic datasets from the developing human cerebral cortex. At the single-cell level, we find once again that HC138 genes are also broadly enriched across inhibitory and excitatory neurons (Fig. 6). To test which neuronal subtype shows greater specificity, we performed conditional enrichment analysis between the neuronal lineage populations and found excitatory neurons to have an independent signal with respect to controlling interneuron signals, while neither enrichment nor significance (*P* = 1 for all) is reached in any neuronal lineage population when controlling for excitatory neurons (*SI Appendix*, Fig. S11). Among excitatory neuron clusters, we find that the HC138 genes are most strongly enriched in visual cortex neurons, which is consistent with recently reported changes in gene expression in the postmortem ASD brain tissue samples (39). When stratified by PPI-defined cluster membership, we find that cluster 1, which harbors many proteins involved in chromatin modification and gene expression regulation, are enriched in progenitor cells and particularly ventral telencephalic neural progenitor cells (“MGE-IPC,” “MGE-div”) and newborn GABAergic neurons (“nIN”). Cluster 2, which includes proteins involved in neuronal communication and ion channel function, are enriched in cortical excitatory and inhibitory neurons, including excitatory neurons of the prefrontal cortex (“EN-PFC”), as previously reported (40), and caudal ganglionic eminence derived GABAergic neurons (“IN-CTX-CGE”). Additional analysis of clusters 3 and 4 revealed potential trends but did not reach statistical significance after multiple test corrections. Genes in cluster 4 (serine/protease phosphorylation), for example, are biased in their expression toward outer radial glia, which are primate-enriched neural stem cells believed to be responsible for the majority of upper cortical layer neurogenesis in humans (41–43), while genes in cluster 3 (insulin receptor function) are enriched in vascular endothelial and mural cells. We performed a similar single-nucleus transcriptomic analysis for the top 10 genes showing an enrichment trend for DNVs in either males or females. Using the HC138 genes as the background gene set, we observed an enrichment signal for intermediate progenitor cells for the female-enriched genes and no such signal for male-enriched genes (*SI Appendix*, Fig. S12).

## Discussion

Here, we present a comprehensive DNV analysis from 15,560 ASD (6,557 from SPARK) and 31,052 DD patients as well as 5,241 unaffected siblings (3,034 from SPARK) using three different statistical models to increase the power to identify risk genes and highlight primary diagnosis, sex, mutation class, and model-specific differences. In the combined NDD group, we identify 615 genes supported by any one or more of the three models as NDD candidates (LC615, union FDR 5%), 237 genes of which show nominal significance by all three models (MC237, intersection FDR 5%), and 138 genes further reaching exome-wide significance by all tests (HC138, intersection FWER 5%). Among the MC237 genes, 27 were additionally identified only in the combined NDD set and not observed when ASD and DD were considered independently (Fig. 2), highlighting the value of combined analyses. For example, *SYNCRIP* (OMIM: 616686), a gene already strongly implicated in NDDs (44, 45), shows one dnMIS and two dnLGD variants in DD patients as well as two dnLGD variants in ASD patients in this dataset; it reaches FDR significance supported by all three models and FWER significance by the CH model when combined but no significance when ASD and DD are analyzed separately. This is consistent with the prevalence of ASD and DD/ID reported in *SYNCRIP*-related disorder. Among the high-confidence genes (HC138), 10 reach exome-wide significance (intersection FWER 5%) only when the ASD and DD probands were combined as a broad NDD group, two genes of which (*PABPC1* and *PSMC5*) represent candidates for further investigation (Fig. 2).

Among the MC237 genes, we identify nine genes with potential associations, and among the HC138 genes with the highest confidence (intersection FWER 5%), we highlight three genes (*MED13*, *NALCN*, and *PABPC1*) reaching exome-wide significance first in this study and two of which with clinical evidence supported the NDD association. *MED13*, a gene that encodes a component of the CDK8-kinase module, has previously been identified through case reports and its phenotype investigated as part of a GeneMatcher collaboration (32). For *NALCN*, Chong and colleagues (33) recently described an autosomal dominant disorder associated with DD and multiple congenital contractures of the face and limbs, extending the phenotype beyond Freeman-Sheldon syndrome to include the impaired cognitive phenotype. Another gene, *PABPC1*, encodes a poly-A RNA binding protein and is thought to play a critical role in the structure of the translation initiation complex (46). To our knowledge, this is one of the first reports of DNV excess in *PABPC1*; however, four DD cases with dnMIS variants clustered in *PABPC1* were reported recently (47), which found that *PABPC1* variants decreased binding affinity to messenger RNA metabolism-related proteins, such as PAIP2, and *Pabpc1* knockdown in mice decreased the proliferation of neural progenitor cells, supporting our finding of *PABPC1* DNV excess in NDDs.

Replication of these findings is important, and the case-control mutational burden analysis of ultra-rare LGD mutation (MAF < 0.01%) using independent SPARK cases (*n* = 21,200) compared to the ExAC nonpsychiatric subset of controls (*n* = 45,376) provided an opportunity to assess some of the new genes at least in the context of autism risk. At a 5% FDR, we found additional support for 52 genes—25 are in the HC138 set, 11 are among MC237, and the remaining 16 correspond to the low-confidence set LC615 genes. As genetic data from whole-exome and whole-genome data continue to grow, especially from families in which complete trios cannot be ascertained, it is likely that such case-control comparisons of ultra-rare mutation will become more and more valuable.

The high-confidence genes (HC138) identified in this study show a consistent pattern of panneuronal expression, which appear to be driven in large part by excitatory neurons. A comparison of single-nucleus transcriptomic data with specific functional PPI networks reveals a more nuanced relationship in which specific functionally related genes clearly associate with specific cellular lineages in the brain. Four of the five PPI networks in this study show differential enrichment in specific lineages ranging from rapidly dividing progenitor cells (cluster 1) and outer radial glial cells (cluster 4) to established excitatory and inhibitory neuronal lineages (cluster 2) to cell types associated with the vascularization and maintenance of the blood–brain barrier (cluster 3). Although not all of these findings are yet statistically significant and will become refined as highly curated cellular-resolution datasets become available, they suggest the possible contribution of these specific cell types to a subset of NDD disease phenotypes and are consistent with observations from studies of large effect size copy-number variations on cortical development (48, 49). If functional networks and definition of cell types and circuits are important targets of future therapeutics, then distinguishing patients with DNVs in these specific genes and further exploration of these PPI networks will be important areas of future research.

Given the locus heterogeneity of ASD and DD, it will be important to continue to expand the exomes and genomes of parent–child trios to identify all of the genes associated with excess DNVs in NDDs. The candidate genes identified here with different levels of significance provide an important resource for further genetic and functional characterization. Severe dnMIS30 or dnLGD variants among the HC138 and MC237 genes account for only 3,029 (6.5%) and 3,683 (7.9%) of the NDD families in this cohort, respectively. Thus, many risk genes are awaiting discovery. Our analyses highlight the value of combining ASD and DD data in increasing power to identify NDD risk genes. While there is ample evidence of DD-specific genes, we find little or no support for ASD-specific genes, although they do identify candidates with trends toward ASD diagnostic enrichment, but none reach statistical significance yet (e.g., *CHD8*, *KDM5B*, *WDFY3*) (22).

There are also several limitations with this study. The size of the DD cohort was double that of the ASD patient cohort and, as a result, favored the discovery of DD-specific genes where the yield of DNVs is greater. The SPARK project and other collaborative initiatives aim to generate exome data from over 50,000 ASD families over the next few years, which will help rectify this imbalance (34). In addition, there is always ascertainment bias, because different cohorts identify probands in different ways. We are likely missing both significantly impaired individuals who obtain independent genetic testing as well as individuals to whom genetic testing may not provide value, as has been observed in schizophrenia (50). Another limitation of this meta-analysis is that not all of the underlying sequence data were available or could be accessed for all published cohorts, leaving only ∼50% of the families that could be reprocessed using the exact same parameters to create a harmonized dataset. Such impediments need to be rectified for the benefit of the research community and the families who contributed their DNA for the purpose of research. Finally, most of the cohorts included in this study originate from exome data in which platform, quality, and coverage vary more widely than genome data. It has been estimated that 5 to 8% of genic regions are inaccessible by earlier exome platforms (51). In the near future, repeating these analyses with genome data or even ultimately generated by long-read sequencing technologies, and also investigating inherited variants, will help identify risk genes or loci (52–54).

In summary, we performed an integrated meta-analysis using three different statistical models on rare DNVs from a large-scale NDD cohort that mainly included ASD and DD probands and unaffected siblings. We identify 615 candidate genes (LC615, union FDR <0.05) and highlight 138 high-confidence genes (HC138, intersection FWER <0.05). We find that NDD genes group into five functional networks where distinct patterns of single-cell expression in the developing brain are suggested. Based on DNV enrichment, we find no evidence of ASD-specific genes in contrast to DD, but we do find clear evidence of genes with sex bias (*n* = 10) as well as genes (*n* = 53) with biases by mutational class (e.g., missense versus loss of function).

## Materials and Methods

### Samples and DNVs.

This study was approved by the University of Washington institutional review board (#STUDY00000383). Raw DNVs were collected or generated from 11 parent–child cohorts (>44,800 families) with exome or genome data available (*SI Appendix*, Table S1). We preferentially retain genome over exome data when both are available. DNVs were identified by analyzing/reanalyzing the underlying sequencing data using the same pipeline when available or directly retrieved from the publications when not. DNVs were annotated using CADD (version 1.3, hg19) and VEP (Ensembl GRCh37 release 94) and restricted to the canonical transcript with the most deleterious annotation. Stringent QC and filtering measures were applied on both samples and DNVs as detailed in the *SI Appendix*. The final integrated set includes a total of 46,612 nonredundant NDD cases with a primary diagnosis of ASD (*n* = 15,560) or DD (*n* = 31,052), and unaffected siblings (*n* = 5,241) (*SI Appendix*, Table S1).

### Statistical Analyses.

De novo enrichment analyses were performed independently for ASD, DD, and the combined NDD groups by using three models: the CH model, denovolyzeR, and DeNovoWEST. We considered two metrics of significance by the union and intersection of three models that corrected by the Benjamini-Hochberg (FDR) and Bonferroni (FWER) methods accounting for the total number of genes tested in each model (18,946 genes in CH model, 19,618 genes in denovolyzeR, and 18,762 genes in DeNovoWEST). We excluded genes that show any significance in the siblings. For each variant category, we required each gene to have more than two DNVs to be considered significant. De novo enrichment analyses in males and females, and the recalled and no-recall subsets, were performed in a similar way. A one-sided Fisher’s exact test was used to test the mutational burden of ultra-rare (MAF < 0.01%) LGD variants between additional independent SPARK exomes (WES2 and WES3, *n* = 21,200) and the ExAC nonpsychiatric control subset (*n* = 45,376). Multiple test correction FDR was performed using the Benjamini-Hochberg method. For the de novo enrichment and mutational burden analyses between males and females, chromosome X was considered to be one copy in males and two copies in females. All of the statistics were calculated using R (version 3.6.2). More details are provided in the *SI Appendix*.

### PPI Analyses and Hub Genes.

The PPI network was assessed using the STRING database with default settings and imported into Cytoscape for downstream analysis. Hub genes (most interacted genes) were assessed using cytoHubba by 12 methods as previously described (37). The hub genes were the top 20 genes supported by at least half of the 12 methods. The PPI clusters were identified by the Markov Cluster Algorithm (MCL, https://micans.org/mcl/). The top three GO functions were selected from rank order of the functional enrichment from the STRING database with default settings. More details are provided in the *SI Appendix*.

### GTEx Brain Expression Evaluation.

The median gene-level transcript expression by tissue was downloaded from GTEx. The average TPM values for LC615 genes (Dataset S6) in the brain were calculated from 13 brain tissues (amygdala, anterior cingulate cortex, caudate, cerebellar hemisphere, cerebellum, cortex, frontal cortex, hippocampus, hypothalamus, nucleus accumbens, putamen, spinal cord, and substantia nigra). The baseline expression levels are defined with the following cutoff: TPM ≥ 1,000, high expression; 1,000 > TPM ≥ 10, medium expression; 10 > TPM ≥ 0.5, low expression; TPM < 0.5, no expression or below cutoff. See more details in the *SI Appendix*.

### Single-Nucleus RNA Expression Analysis.

The dataset includes single-nucleus transcriptomes from 49,495 nuclei across multiple human cortical areas. Nuclei were sampled from postmortem and neurosurgical (middle temporal gyrus only) donor brains and expression was profiled with SMART-Seq version 4 RNA-seq. Unsupervised clustering with Seurat identified 120 distinct transcriptomic clusters, including 54 GABAergic (inhibitory) neuronal, 56 glutamatergic (excitatory) neuronal, and 10 nonneuronal cell types. Heatmaps were constructed of log-normalized trimmed mean expression (excluding the 25% lowest and 25% highest expression values), log_2_(CPM + 1) (CPM, counts per million), of NDD and control gene sets across cell types. Genes were ordered by the number of cell types with trimmed mean expression > 1. The *SI Appendix* has more details.

### Tissue and Cell-Type-Specific Expression of Significant Genes.

scRNA-seq data were pulled from the University of California, Santa Cruz Cell Browser (cells.ucsc.edu/?ds=cortex-dev) and CPM counts were quasinormalized into unique molecular identifiers (UMIs) using quminorm (https://github.com/willtownes/quminorm). Cells were then regrouped by their broad parent cell types, with unknown cell types filtered out. SCTransform (https://github.com/ChristophH/sctransform) was used to normalize the UMI counts from quminorm. The corrected counts from SCTransform were used as input into expression weighted cell-type enrichment (EWCE) following the default parameters with two levels of annotations based on clusters and clusters split by sex. Bootstrapping parameters in EWCE were as follows: 10,000 repetitions with the LC615 genes as background in the unconditional enrichment and HC138 for the controlled experiments. Cluster-specific analysis within the most stringent gene set and top sex-specific genes used the HC138 genes as background. Online TSEA and CSEA tools were used to determine the enrichment of expression across brain regions and cell types (55). The expression among these tissues was compared using Fisher’s exact tests and followed by Benjamini-Hochberg correction (*SI Appendix*).

### Assessment of Gene Intolerance Scores.

We applied the ExAC-based residual variation intolerance score (RVIS) and missense constraint score (mis_Z score), as well as the gnomAD-based LOEUF score. A Wilcoxon two-sample test was performed in R (version 3.6.2) using the wilcox.test function. More details are provided in the *SI Appendix*.

## Supplementary Material

Supplementary File

Supplementary File

Supplementary File

Supplementary File

Supplementary File

Supplementary File

Supplementary File

Supplementary File

Supplementary File

Supplementary File

Supplementary File

Supplementary File

## Data Availability

The underlying genomic and phenotypic data in the recalled subset are available from the following resources: the sequencing and phenotype data for the Simons Simplex Collection (SSC) cohort (https://www.sfari.org/resource/simons-simplex-collection/) are available to approved researchers at SFARI Base (https://base.sfari.org, SFARI_SSC_WGS_p, SFARI_SSC_WGS_1, and SFARI_SSC_WGS_2). Genome data from the SAGE samples are available at dbGaP (phs001740.v1.p1) (56). Family-level FreeBayes and GATK VCF files for SSC and SAGE samples are available at dbGaP (phs001874.v1.p1) (57) and also at SFARI Base (SFARI_SSC_WGS_2a). The genomic and phenotypic data for the SPARK study (https://www.sfari.org/resource/spark/) are available at SFARI Base (SFARI_SPARK_pilot, SFARI_SPARK_WES_1, SFARI_SPARK_WES_2, and SFARI_SPARK_WES_3). The genomic and phenotypic data for the DDD study are available from the European Genome-phenome Archive (EGA, https://ega-archive.org) under study EGAS00001000775, (58). The DNVs in the no-recall subset are retrieved from each of the corresponding publications (*SI Appendix*, Table S2). The final harmonized DNVs, from both the recalled and no-recall subsets, used in the study are available in Dataset S1. Codes used to generate the figures are available at https://github.com/tianyunwang/ndd_meta_2022 (59). Web URLs and abbreviations are provided in the *SI Appendix*.
