# Supplementary material for: Integrated gene analyses of de novo variants from 46,612 trios with autism and developmental disorders
Source: Proc Natl Acad Sci U S A. 2022 Nov 11;119(46):e2203491119. doi: 10.1073/pnas.2203491119 (PMC9674258; doi:10.1073/pnas.2203491119)
Supplement: Supplementary File [file pnas.2203491119.sapp.pdf]

SI Appendix for

# Integrated gene analyses of *de novo* variants from 46,612 trios with autism and developmental disorders

Wang et al.

**This PDF file includes:**

Dataset Titles
Web URLs
Abbreviations
Supplementary Methods
Tables S1 to S5
Figures S1 to S15
Supplementary Analyses
Supplementary References
The SPARK Consortium Authors

**Datasets S1 to S14 are provided individually in separate XLSX files:**

Dataset S1. Integrated coding DNVs.

Dataset S2. *De novo* enrichment analysis in siblings (n = 5,241).

Dataset S3. *De novo* enrichment analysis in NDD patients (n = 46,612).

Dataset S4. *De novo* enrichment analysis in DD patients (n = 31,052).

Dataset S5. *De novo* enrichment analysis in ASD patients (n = 15,560).

Dataset S6. Significance overview of the LC615 NDD candidate genes.

Dataset S7. Intolerance score and DNV in ASD versus DD patients for all significant genes.

Dataset S8. Genes with significant burden of dnLGD and dnMIS variants in NDD patients (n = 46,612).

Dataset S9. *De novo* enrichment analysis in male patients (n = 29,704).

Dataset S10. *De novo* enrichment analysis in female patients (n = 16,530).

Dataset S11. Top hub genes by cytoHubba.

Dataset S12. *De novo* enrichment analysis in recalled subset (n = 19,375).

Dataset S13. *De novo* enrichment analysis in no-recall subset (n = 27,237).

Dataset S14. Duplicate regions used in DNV filtering.

**Web URLs**

Software, database resources, and custom algorithms central to the research in this study are publicly available as follows:

denovo-db: http://denovo-db.gs.washington.edu/

SPARK: https://sparkforautism.org

Ensembl VEP (GRCh37): http://grch37.ensembl.org/Homo_sapiens/Tools/VEP/

CADD score: https://cadd.gs.washington.edu/

FreeBayes: https://github.com/ekg/freebayes

CH model: https://github.com/tianyunwang/CH-model

denovolyzeR: https://github.com/jamesware/denovolyzeR

DeNovoWEST: https://github.com/queenjobo/DeNovoWEST

quminorm: https://github.com/willtownes/quminorm

SCTransform: https://github.com/ChristophH/sctransform

UCSC Cell Browser: https://cells.ucsc.edu

TSEA tool: http://genetics.wustl.edu/jdlab/tsea/

CSEA tool: http://genetics.wustl.edu/jdlab/csea-tool-2/

RVIS score: http://genic-intolerance.org/

STRING: https://string-db.org/

Cytoscape: https://cytoscape.org/

cytoHubba: http://apps.cytoscape.org/apps/cytohubba

ProteinPaint: https://proteinpaint.stjude.org/

DDG2P: https://www.ebi.ac.uk/gene2phenotype

SFARI Gene: https://gene.sfari.org/

SFARI Base: http://base.sfari.org

EGA: https://ega-archive.org/

OMIM: https://omim.org

gnomAD: https://gnomad.broadinstitute.org/

**Abbreviations**

DNV: *de novo* variant

NDD: Neurodevelopmental disorder

ASD: Autism spectrum disorder

DD: Developmental disorder

ID: Intellectual disability

CCDG: Centers for Common Disease Genomics

SFARI: Simons Foundation Autism Research Initiative

SPARK: Simons Foundation Powering Autism Research for Knowledge

SSC: Simons Simplex Collection

ASC: Autism Sequencing Consortium

DDD: Deciphering Developmental Disorders

RUMC: Radboud University Medical Center

CADD score: combined annotation dependent depletion score

LGD: likely gene-disruptive

dnLGD: *de novo* LGD variant

dnSYN*: de novo* synonymous variant

dnMIS: *de novo* missense variant

dnMIS30: *de novo* missense variant with CADD score greater than 30

FDR: False discovery rate

FWER: Family-wise error rate

PPI: Protein-protein interaction

CSEA: Cell-type-specific expression analysis

TSEA: Tissue-specific expression analysis

DDG2P: Development Disorder Genotype - Phenotype Database

OMIM: Online Mendelian Inheritance in Man

LC615 genes: The most comprehensive set of 615 genes with lowest confidence based on the union FDR 5% significance by one or more of three models, which includes the MC237 and HC138 genes.

MC237 genes: 237 genes with moderate confidence based on the intersection FDR 5% significance by all three models—it is a subset of the LC615 genes, but includes all HC138 genes.
HC138 genes: The most stringent of 138 genes with the highest confidence based on the intersection FWER 5% significance by all three models—it is a subset of the HC237 and LC615 genes.

## Supplementary Methods

**Cohorts and samples.** This study was approved by the University of Washington Institutional Review Board #STUDY00000383, Genetics Consortium Repository. Informed consent was obtained from all subjects by each of the corresponding study cohort. We collected eight exome and three genome parent–child cohorts (>100 trios of each), which include over 44,800 families (Table S1). We preferentially retain genome over exome data for cohorts that have both types of data available. We only included the Centers for Common Disease Genomics (CCDG) genomes for Simons Simplex Collection (SSC) samples. For cohorts with continuous publications, like for the Autism Sequencing Consortium (ASC), Deciphering Developmental Disorders (DDD), and Radboud University Medical Center (RUMC) samples, we only included the final samples from their latest study with potential sample duplicates removed. We also excluded any potential overlaps in the literature; for example, we excluded all SSC samples used in the ASC paper[1] for potential redundancy with the CCDG SSC genomes. After all those control measures, we also ran KING[2] (v1.4) for samples with the underlying sequencing data available for further detection of potential sample overlap. KING uses identical by state (IBS) to estimate pairwise relatedness between samples; any samples with a kinship value >0.35 were considered as potential sample duplicates. We first checked if the potential duplicates were known as monozygotic twin pairs or known duplicates within a cohort (some individuals had both blood and cell line DNA sequenced for QC purposes). Only one sample was retained from each duplicate pair in downstream analyses—blood DNA was preferred if both blood and cell line sequencing data were available.

**DNV discovery and integration.** DNVs were identified by analyzing/reanalyzing the underlying sequencing data wherever available for the five cohorts using the same pipeline. Specifically, DNVs were harmonized by reanalyzing 70,172 samples (46.5%), including 24,520 families within two categories. First, for ASD cohorts with genome sequencing data from the CCDG study, including SSC and the Study of Autism Genetics Exploration (SAGE), raw single-nucleotide variant/insertion or deletion (SNV/indel) variants were called (on hg38) independently using four different callers: GATK[3], FreeBayes[4], Platypus[5], and Strelka2[6]. Downstream DNV discovery was based on genotype, which is required only if the offspring has the alternative allele (with genotype as 0/1 or 1/1) but is not observed in either of the parents (with genotype as 0/0). Candidate DNVs

needed to have the support of at least two of the four callers; and then variants from Platypus with a filter of LowGQX or NoPassedVariantGTs were removed, and Strelka2 variants had to have the filter field equal to PASS. For variants on the X chromosome, we separately considered variants in the pseudoautosomal regions (chrX:10000-2781479, chrX:155701382-156030895, hg38) and the X/Y duplicative transposed region (chrX:89201803-93120510, hg38). Candidate DNVs were then converted to hg19 for downstream integration. Second, for part of other exome cohorts, including SPARK_pilot, SPARK_WES_1, and DDD, raw SNV/indel variants were called (on hg19) independently using GATK and FreeBayes. Downstream DNV discovery was based on genotype as applied in genome cohorts. Candidate DNVs needed to have the support from both callers, which is the intersection set by GATK and FreeBayes. Beyond the above measures, we also applied the following variant-level filters: allele balance (AB = 0 in both parents, and AB > 0.25 in the child), read depth (DP > 9 for all family members), child genotype quality (GQ > 20 by both GATK and FreeBayes). For the rest cohorts included in studies with no underlying sequencing data available, DNVs were collected from each corresponding publication. DNVs on hg38 were first converted to hg19; the final integrated DNVs were all on hg19. To combine DNVs between exome and genome datasets, we restricted DNVs to a well-covered coding region[7] (average DP > 20X) generated by accessing the exome data from the SSC, SPARK, and DDD cohorts. We also removed all DNVs in the segmental duplication regions, recent repeat and low-complexity regions, or centromeric and telomeric regions[8] (Table S17). We excluded variants in a homopolymer A or T of length 10 or greater, and the variants with a reference or alternative allele with greater than 10 bp, to remove potential sequencing errors. Beyond all the above filtering and sample duplicate exclusions, we further excluded samples with more than 10 coding DNVs as outliers and removed specific DNVs that were observed in more than five different unrelated individuals for frequency control. All of the above strict measures yielded a total of 46,612 nonredundant NDD cases with a primary diagnosis of ASD (n = 15,560) or DD (n = 31,052), and also unaffected siblings (n = 5,241) in the integrated *de novo* enrichment analysis (Table S1). To ensure uniformity, the same versions of CADD score (v1.3) and VEP annotation (Ensembl GRCh37 release 94) were applied, and the analysis was restricted to the canonical transcript with the most deleterious annotation.

**Statistical analyses.** *De novo* enrichment analyses were performed independently for ASD, DD, and combined NDD samples by using three statistical models: the CH model, denovolyzeR, and DeNovoWEST. All three methods apply their own underlying variant rate estimates (denovolyzeR and DeNovoWEST use the same rate while the CH model is different) to generate the prior probabilities for observing a specific number and class of mutations for a given gene. Briefly, the CH model estimates the number of expected DNVs by incorporating locus-specific transition, transversion, and indel rates and chimpanzee–human coding sequence divergence and the gene length; while denovolyzeR estimates mutation rates based on trinucleotide context and incorporates exome depth, mutational biases such as CpG hotspots, and divergence adjustments based on macaque–human comparisons. DeNovoWEST scores all classes of coding variants on a unified severity scale based on the empirically estimated positive predictive value of being pathogenic and incorporates a gene-based weighting derived from the deficit of protein-truncating variants in the general population, further combining missense enrichment by a clustering test. Default parameters were used for all three methods with some minor adjustments, such as in the process of weight creation in DeNovoWEST, fewer numbers of CADD bins for missense and nonsense variants were used for ASD samples (three bins), versus in the DD and combined NDD group where seven bins were used for both, due to the sample size differences as suggested[6]. The expected mutation rate of 1.8 DNVs per exome was set to the CH model as an upper bound baseline. Siblings were also analyzed similarly using the CH model and denovolyzeR, but not run for DeNovoWEST due to the small sample size. We applied two metrics of significance with the union and intersection of three models: first is the FDR significance, the significance threshold ($q < 0.05$) was corrected exome-wide using the Benjamini–Hochberg method by accounting for the total number of genes in each model (18,946 genes in CH model, 19,618 genes in denovolyzeR, and 18,762 genes in DeNovoWEST); the second metric is a more stringent FWER significance, for which we applied exome-wide Bonferroni multiple-testing correction considering both the largest number of genes among three models ($n = 19,618$) and the total of tests per gene across the three models. For probands, FWER 5% significance threshold ($p < 3.64e-07$) was corrected by the Bonferroni method for 19,618 genes and seven tests in the analysis (dnLGD, dnMIS, and dnMIS30 variants in the CH model, dnLGD and dnMIS variants in denovolyzeR, and dnLGD and dnMIS variants in DeNovoWEST). For siblings, FWER 5% significance threshold ($p < 5.09e-07$)

was corrected for 19,618 genes but only five tests in the CH model and denovolyzeR. We excluded genes that show any significance in the siblings from the counting of significant genes in probands (ASD, DD, and NDD). For each variant category, we required each gene to have more than two DNVs to be considered as significant. *De novo* enrichment analyses in the males and females, and the recalled and no-recall subsets, were performed in the similar way. For the *de novo* enrichment and mutational burden analyses comparing males and females, chromosome X was considered as one copy in males and two copies in females.

As for the case–control analysis for the LC615 genes, we identified ultra-rare (MAF < 0.01%) LGD variants from two independent autism cohorts: SPARK_WES_2 (10,876 families, 16,604 samples, and 11,912 cases) and SPARK_WES_3 (9,941 families, 16,779 samples, and 9,288 cases), compared to the ExAC non-psych subset (n = 45,376). Variants from SPARK exomes were first filtered based on read depth (>10X) and genotype quality (GQ > 20). We restricted the variants to be within the intersected region of both the SPARK exome capture and ExAC reliably called regions. Variants were only considered if they could be called in >90% of samples with >10X read depth in ExAC samples to control for coverage balance. A one-sided Fisher's exact test was used to test for case–control mutational burden analysis between SPARK exomes (WES2 and WES3, n = 21,200) and the ExAC non-psych subset (n = 45,376). Multiple test correction FDR was performed by the Benjamini–Hochberg method. Statistics were calculated using R (version 3.6.2).

**Enrichment analyses in recalled and no-recall subsets.** We also performed same enrichment analyses using the three models in parallel for those two subsets. We identified 323 FDR (132 FWER) significant genes in the recalled subset and 389 FDR (174 FWER) significant genes in the no-recall subset (Tables S15-S16). For those FDR-significant genes, of which 87.3% (282/323) in the recalled subset and 90.0% (350/389) in the no-recall subset overlap with the LC615 genes in combined NDD group (Figure S13), suggests consistent results in both subsets after data harmonization. However, there are 12 exclusively significant genes in the no-recall subset and one exclusively significant gene (*PABPC1*) in the recalled subset among the HC138 genes in combined NDD group. A closer look found those 12 genes were also reported as significant genes in a recent study[9], and the significant signal was driven by DNVs almost exclusively from GeneDx,

for which the raw data is not available for recalling. For example, all DNVs in three genes (*ZEB2*, *PDHA1*, and *SLC2A1*) are from GeneDx probands (n = 18,783) and none among the other five cohorts (n = 8,454) in the no-recall subset (Figure S13). This is consistent with the original study where the majority of the DNVs are from GeneDx cohort, with very few from the DDD and RUMC samples. This draws attention to the significance of such genes, where the significance signal is mostly driven by DNVs from a single cohort and no underlying data is available for further QC reanalyzing.

**PPI analyses and hub genes.** The PPI network was assessed by searching Multiple Proteins by Names using the online STRING database with default settings. The interaction result was exported as a TSV file and then imported into Cytoscape software for downstream analysis. We used cytoHubba to identify top hub genes (most interacted genes); cytoHubba provides the analyzed results computed by 12 methods, including Degree, clustering coefficient, Edge Percolated Component (EPC), Maximum Neighborhood Component (MNC), Density of Maximum Neighborhood Component (DMNC), Maximal Clique Centrality (MCC), and centralities based on shortest paths, such as Bottleneck (BN), EcCentricity, Closeness, Radiality, Betweenness, and Stress, as previously described[10]. The top 20 genes were supported by the most, and at least half, of the models as top hub genes. The PPI clusters were identified by the Markov Cluster Algorithm (MCL, https://micans.org/mcl/). The top three GO functions were selected from rank order of the functional enrichment from STRING database with default settings.

**GTEx brain expression evaluation for LC615 genes.** The gene and transcript expression in GTEx are shown in transcripts per million (TPM). The median gene-level TPM by tissue dataset GTEx_Analysis_2017-06-05_v8_RNASeQCv1.1.9_gene_median_tpm.gct.gz (https://storage.googleapis.com/gtex_analysis_v8/rna_seq_data/GTEx_Analysis_2017-06-05_v8_RNASeQCv1.1.9_gene_median_tpm.gct.gz) was downloaded from the GTEx website (https://gtexportal.org/home/datasets). The average TPM of brain was calculated from TPM values of 13 brain tissues (amygdala, anterior cingulate cortex, caudate, cerebellar hemisphere, cerebellum, cortex, frontal cortex, hippocampus, hypothalamus, nucleus accumbens, putamen, spinal cord, substantia nigra), which was provided in Table S8. The baseline expression levels

are defined with the following cutoff: TPM ≥ 1000, high expression; 1000 > TPM ≥ 10, medium expression; 10 > TPM ≥ 0.5, low expression; TPM < 0.5, no expression or below cutoff.

**Single-nucleus RNA expression analysis.** The dataset includes single-nucleus transcriptomes from 49,495 nuclei across multiple human cortical areas. Individual cortex layers were dissected from tissues covering the middle temporal gyrus (MTG), anterior cingulate cortex (ACC; also known as the ventral division of medial prefrontal cortex, A24), primary visual cortex (V1C), primary motor cortex (M1C), primary somatosensory cortex (S1C), and primary auditory cortex (A1C) derived from human brain. Nuclei were dissociated and sorted using the neuronal marker NeuN. Nuclei were sampled from postmortem and neurosurgical (MTG only) donor brains and expression was profiled with SMART-Seq v4 RNA-seq. The data are available from the Allen Institute for Brain Science website for analysis (https://celltypes.brain-map.org/rnaseq/human_ctx_smart-seq) and download (https://portal.brain-map.org/atlases-and-data/rnaseq/human-multiple-cortical-areas-smart-seq). Unsupervised clustering with Seurat identified 120 distinct transcriptomic clusters, including 54 GABAergic (inhibitory) neuronal, 56 glutamatergic (excitatory) neuronal, and 10 non-neuronal cell types. Heatmaps were constructed of log-normalized trimmed mean expression (excluding the 25% lowest and 25% highest expression values), $\log_2(CPM + 1)$, of NDD and control gene sets across cell types. Genes were ordered by the number of cell types with trimmed mean expression > 1. For each cell class (GABAergic and glutamatergic neurons and non-neuronal cells), the number of cell types with trimmed mean expression > 1 for NDD risk genes and control genes were quantified and visualized as empirical cumulative distributions (Figure S8). A Kolmogorov–Smirnov test was used to reject the null hypothesis that the cell type count distributions were the same between each gene set and the control DNV gene set. P-values were Bonferroni-corrected for multiple testing. Similarly, for each cell subclass (e.g., SST interneurons or L6b excitatory neurons), the trimmed mean expression levels of NDD risk genes and control genes were quantified and visualized as empirical cumulative distributions (Figure S7). A Kolmogorov–Smirnov test was used to reject the null hypothesis that the expression distributions were the same between each gene set and the control DNV gene set. P-values were Bonferroni corrected for multiple testing and $-\log_{10}$-transformed and visualized as a heatmap with columns corresponding to cell

subclasses ordered by Ward's clustering and rows corresponding to gene sets. Full names and detailed descriptions of each cell subtype in the heatmap (Figure 6) are: GABAergic interneuron (LAMP5: LAMP5 expressing GABAergic neuron, PAX6: PAX6 expressing GABAergic neuron, PVALB: PVALB expressing GABAergic neuron, SST: SST expressing GABAergic neuron; VIP: VIP expressing GABAergic neuron); Glutamatergic neuron (IT: intratelencephalic neuron, L4 IT: Layer 4 intratelencephalic neuron, L5 ET: Layer 5 extratelencephalic neuron, L5/6 IT Car3: Layer 5/6 intratelencephalic neuron that selectively expresses Car3, L5/6 NP: Layer 5-6 Near-projecting neuron, L6 CT: Layer 6 corticothalamic neuron, L6b: Layer 6b neuron); and non-neuronal cell (astrocyte, microglia, oligodendrocyte, OPC: oligodendrocyte progenitor cell).

**Tissue and cell-type-specific expression of significant genes.** scRNA-seq data were pulled from UCSC Cell Browser (http://cells.ucsc.edu/?ds=cortex-dev) and CPM counts were quasi normalized unto unique molecular identifies (UMI) using quminorm (https://github.com/willtownes/quminorm). Cells were then regrouped by their broad parent cell types with unknown cell types filtered out. SCTransform (https://github.com/ChristophH/sctransform) was used to normalize the UMI counts from quminorm. The corrected counts from SCTransform were used as input into expression weighted cell-type enrichment (EWCE) following the default parameters with two levels of annotations based on clusters and clusters split by sex. Bootstrapping parameters in EWCE: 10000 repetitions with the LC615 genes as background in the unconditional enrichment and HC138 for the controlled experiments. Cluster-specific analysis within the most stringent gene set and top sex specific genes (10 for female and 10 for male) used the HC138 genes as background. Online TSEA and CSEA tools were used to determine the enrichment of expression across brain regions and cell types[11]. The expression among these tissues was compared using Fisher's exact tests and followed by Benjamini–Hochberg correction. The significance is calculated from the bootstrap cell-type enrichment test. The test takes a gene list and scRNA-seq data and determines the probability of enrichment and fold changes for each cell type. The proportion of expression in each cell type is calculated as a matrix for each gene, then summed to get the total expression in each cell type across the whole gene list. Thus, for a gene list indexed by *X*, we calculate the average expression in the cell type. This calculation is repeated for randomly generated gene lists

sampled without replacement from background genes controlled for gene length as the target gene list. The probability of cellular enrichment is then calculated based on the number of bootstrapped gene lists with higher cell-type-specific expression than the target list. Where probabilities are stated for gene list enrichments, all p-values stated are adjusted for multiple testing. The signature score was calculated using the AddModuleScore in Seurat (https://satijalab.org/seurat/), the function takes in a list of gene groups, which corresponds here to HC138 genes, with the background of all genes in the scRNA-seq data but not including the HC138 genes. The exact calculation followed by taking average expression levels of each program (cluster) on a single-cell level, subtracted by the aggregated expression of control feature sets. All analyzed features are binned based on averaged expression, and the control features are randomly selected from each bin.

**Assessment of gene intolerance scores.** To assess a gene's intolerance to variation, we applied the ExAC-based residual variance to intolerance score (RVIS) and missense constraint scores (mis_Z scores), as well as the gnomAD-based "loss-of-function observed/expected upper bound fraction" (LOEUF). The LOEUF score is a conservative estimate of the observed/expected ratio based on the upper bound of a Poisson-derived confidence interval around the ratio. It ranges from 0 to 2, with lower LOEUF scores indicating stronger selection against predicted loss-of-function variation in a given gene, and a cut-off value is suggested as 0.35. The mis_Z score indicates a gene's intolerance to missense variants, positive scores indicate more constraint, and negative scores indicate less constraint. A greater Z-score indicates more intolerance to the class of variation. RVIS was based on ExAC v2 release 2.0 (accessed: March 15$^{th}$, 2017). As of this release, we used CCDS release 20 and Ensembl release 87 annotations. The score was converted into percentile by ranking all genes from most intolerant to least. For example, percentile of 1% means the gene is amongst the top 1% of the most intolerant genes. A Wilcoxon two-sample test was performed in R (versions 3.6.2) with the wilcox.test function.

## Table S1. DNV cohorts in this study.

| Group | Cohort | Samples | Proband | Male | Female | Sibling | Male | Female | NGS | Individual Sex info | Study |
|---|---|---|---|---|---|---|---|---|---|---|---|
| ASD | SPARK_WES_1 | 27,256 | 6,557 | 5,229 | 1,328 | 3,034 | 1,551 | 1,483 | WES | Known | This study, Zhou2022[12], and Fu2022[13] |
| | SSC | 8,757 | 2,299 | 1,989 | 310 | 1,860 | 879 | 981 | WGS | Known | CCDG_SSC[14] |
| | SAGE | 547 | 202 | 161 | 41 | - | - | - | WGS | Known | CCDG_SAGE[15] |
| | SPARK_pilot | 1,379 | 465 | 377 | 88 | - | - | - | WES | Known | Feliciano2019[16] |
| | MSSNG | 4,174 | 1,613 | 1,268 | 345 | - | - | - | WGS | Known | Yuen2017[17] |
| | ASC | 12,123 | 4,046 | 3,263 | 783 | 347 | 171 | 176 | WES | Known | Satterstrom2020[1] |
| | JASD | 786 | 262 | 191 | 71 | - | - | - | WES | NA | Takata2018[18] |
| | ACE | 348 | 116 | 98 | 18 | - | - | - | WES | NA | Chen2017[19] |
| | **Sub-total** | **55,370** | **15,560** | **12,576** | **2,984** | **5,241** | **2,601** | **2,640** | | | |
| DD | DDD13K | 32,233 | 9,852 | 5,655 | 4,197 | - | - | - | WES | Known | Kaplanis2020[9] |
| | GeneDx | 56,367 | 18,783 | 10,385 | 8,398 | - | - | - | WES | Known | Kaplanis2020[9] |
| | RUMC | 7,254 | 2,417 | 1,377 | 1,040 | - | - | - | WES | Known | Kaplanis2020[9] |
| | **Sub-total** | **95,854** | **31,052** | **17,417** | **13,635** | **-** | **-** | **-** | | | |
| **NDD** | **Total** | **151,224** | **46,612** | **29,993** | **16,619** | **5,241** | **2,601** | **2,640** | | | |

Cohorts are organized into three groups based on primary phenotype (ASD, DD, and NDD). The number of samples in each cohort are total parent–child samples pre data harmonization. The affected (proband) and unaffected (sibling) counts are samples used in the meta-analysis after data QC and harmonization.

## Table S2. DNV rate across cohorts.

| Subset | Phenotype | Cohort | Probands | dnLGD | rate | dnMIS | rate | dnSYN | rate | DNV | rate |
|---|---|---|---|---|---|---|---|---|---|---|---|
| **recalled** | ASD | SPARK_WES_1 | 6,557 | 658 | 0.10 | 4,160 | 0.63 | 1,891 | 0.28 | 6,709 | 1.02 |
| | ASD | SSC | 2,299 | 261 | 0.11 | 1,273 | 0.55 | 442 | 0.19 | 1,976 | 0.86 |
| | ASD | SAGE | 202 | 17 | 0.08 | 104 | 0.51 | 27 | 0.13 | 148 | 0.73 |
| | ASD | SPARK_pilot | 465 | 58 | 0.12 | 280 | 0.60 | 113 | 0.24 | 451 | 0.97 |
| | DD | DDD13K | 9,852 | 1,624 | 0.16 | 6,571 | 0.67 | 2,296 | 0.23 | 10,491 | 1.06 |
| | | **Sub-total** | **19,375** | **2,618** | **0.14** | **12,388** | **0.64** | **4,769** | **0.24** | **19,775** | **1.02** |
| **no-recall** | ASD | ASC | 4,046 | 451 | 0.11 | 2,349 | 0.58 | 814 | 0.20 | 3,614 | 0.89 |
| | ASD | MSSNG | 1,613 | 160 | 0.10 | 822 | 0.51 | 318 | 0.20 | 1,300 | 0.81 |
| | ASD | JASD | 262 | 44 | 0.17 | 133 | 0.51 | 36 | 0.14 | 213 | 0.81 |
| | ASD | ACE | 116 | 12 | 0.10 | 66 | 0.57 | 11 | 0.09 | 89 | 0.77 |
| | DD | GeneDx | 18,783 | 2,903 | 0.15 | 12,575 | 0.67 | 3,961 | 0.21 | 19,439 | 1.03 |
| | DD | RUMC | 2,417 | 404 | 0.17 | 1,442 | 0.60 | 507 | 0.21 | 2,353 | 0.97 |
| | | **Sub-total** | **27,237** | **3,974** | **0.14** | **17,387** | **0.64** | **5,647** | **0.21** | **27,008** | **0.99** |
| **All** | **ASD+DD** | **Total** | **46,612** | **6,592** | **0.14** | **29,775** | **0.64** | **10,416** | **0.22** | **46,783** | **1.00** |

| Cohort | Siblings | dnLGD | rate | dnMIS | rate | dnSYN | rate | DNV | rate |
|---|---|---|---|---|---|---|---|---|---|
| SPARK_WES_1, SSC, ASC | 5,241 | 329 | 0.06 | 2,999 | 0.57 | 1,290 | 0.25 | 4,618 | 0.88 |

The number of DNVs per proband was calculated for each cohort after data harmonization with the same filtering criteria. The DNV rate is consistent across cohorts as well as across the recalled and no-recall subset cohorts. This table corresponds to Figure S1.

**Table S3. DNV comparison in ASD and DD patients for LC615 NDD candidate genes.**

| Significant genes with non-synonymous DNVs | dnLGD | dnMIS | dnMIS30 | DNVs |
|---|---|---|---|---|
| In ASD only | 42 | 27 | 32 | 13 |
| In DD only | 152 | 158 | 206 | 118 |
| In neither | 245 | 10 | 216 | 0 |
| In both | 176 | 420 | 161 | 484 |
| ASD > DD | 56 | 138 | 58 | 149 |
| ASD < DD | 120 | 282 | 103 | 335 |

A comparison of non-synonymous DNVs, broken down by mutation class, in ASD (n = 15,560) and DD (n = 31,052) patients was performed for all LC615 candidate risk genes. For genes with DNVs in both ASD and DD patients ("In both"), the DNV ratio was further compared with sample size considered for calculation. "ASD > DD" means genes with relatively more DNVs in ASD than DD patients; "ASD < DD" means genes with relatively less DNVs in ASD than DD patients. In the cohort, 78.7% (484/615) of the genes have DNVs in both ASD and DD samples; more genes have high frequency of DNVs in DD (335 genes) than in ASD (149 genes) patients; and more genes have DNVs in DD (118 genes) than in ASD (13 genes) patients.

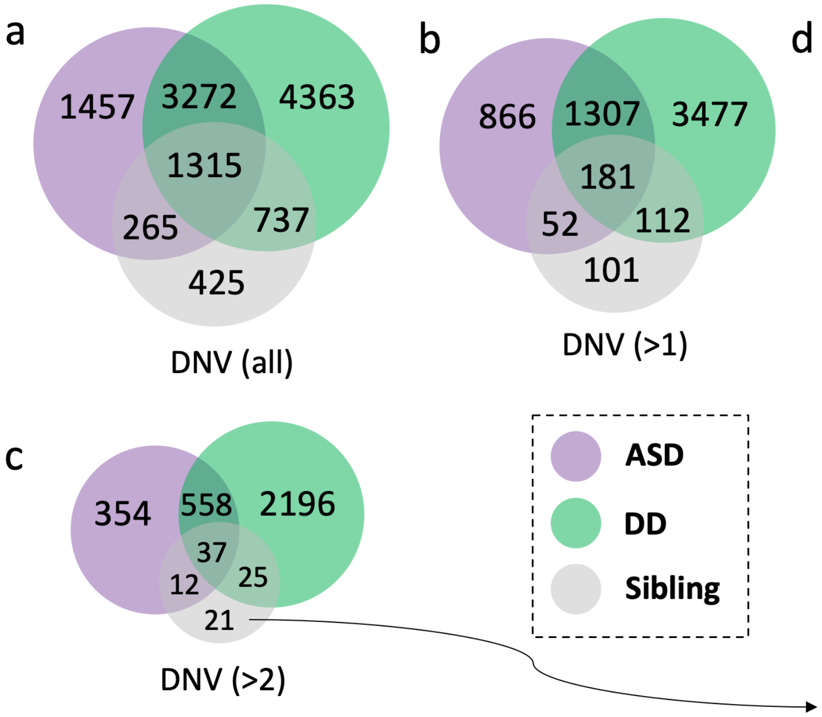


d DNVs and significance in siblings

| Gene | dnLGD | dnMIS | FDR 5% |
|---|---|---|---|
| *CLEC2D* | 0 | 3 | CH&DR (dnMIS) |
| *CEP20* | 0 | 3 | DR (dnMIS) |
| *FAIM* | 0 | 3 | |
| *ADAM18* | 1 | 2 | |
| *CETP* | 0 | 3 | |
| *CFAP54* | 0 | 3 | |
| *DDX25* | 0 | 3 | |
| *ERBB4* | 0 | 3 | |
| *IPO11* | 0 | 3 | |
| *KLHL21* | 0 | 3 | |
| *MYBL2* | 0 | 3 | |
| *NLRP5* | 0 | 3 | |
| *NMI* | 0 | 3 | |
| *PARD3* | 0 | 3 | |
| *PDZRN3* | 1 | 2 | |
| *RABGAP1* | 1 | 2 | |
| *SPOCK3* | 0 | 3 | |
| *THAP9* | 0 | 3 | |
| *TMCC3* | 0 | 3 | |
| *TRIM17* | 0 | 3 | |
| *YIF1A* | 1 | 2 | |

**Figure S1. Overlap of genes with non-synonymous DNVs across phenotype groups.** The majority of genes (82.8%, 2,047/2,472) with DNVs in siblings also have DNVs in probands. There are **(a)** 425 genes (all DNVs), **(b)** 101 genes (DNV > 1), and **(c)** 21 genes (DNV > 2) with DNVs only in siblings at different filtering levels of DNV counts. **(d)** Most of the 21 genes with DNVs (n > 2) exclusively in siblings carry only dnMIS variants, except four genes (*ADAM18*, *PDZRN3*, *RABGAP1*, and *YIF1A*) with one dnLGD variant. Only two genes (*CLEC2D* and *CEP20*) show an excess of dnMIS variants in siblings with FDR at 5% (q-value < 0.05, DNV count > 1).

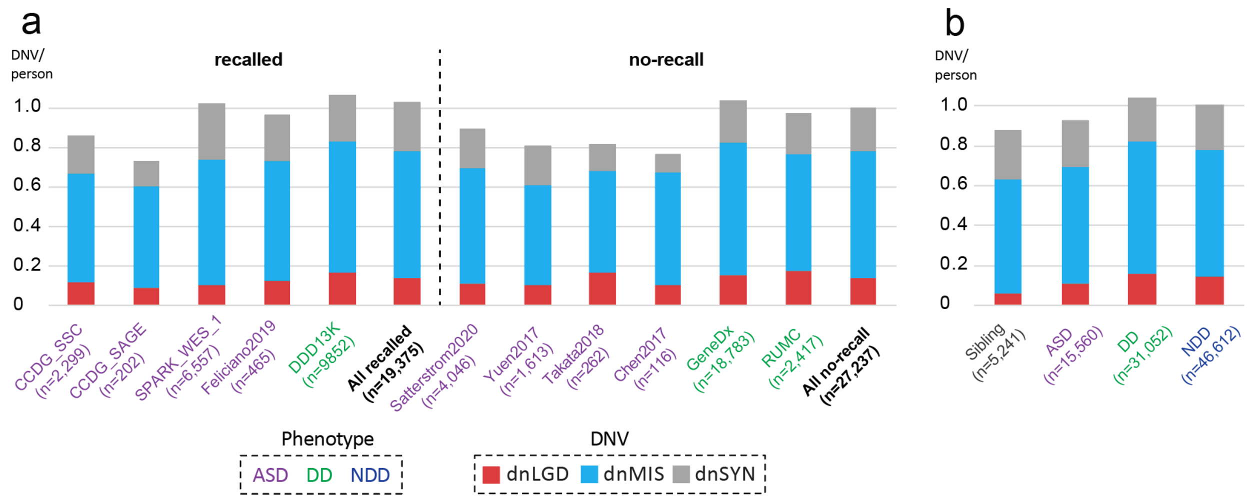


**Figure S2. DNV rate across cohorts.** The number of DNVs per person was calculated for **(a)** each individual cohort and **(b)** each phenotype group after data harmonization with the same filtering criteria applied. The DNV rate is consistent across large cohorts as well as across the cohorts with (recalled) and without (no-recall) reanalysis of the underlying sequencing data. The numbers of probands are also shown with color indicating the phenotype (ASD in purple, DD in green, and NDD in dark blue). Note, the bar plot indicates the number of DNVs per person: they are absolute values and no statistics or distributions are involved.

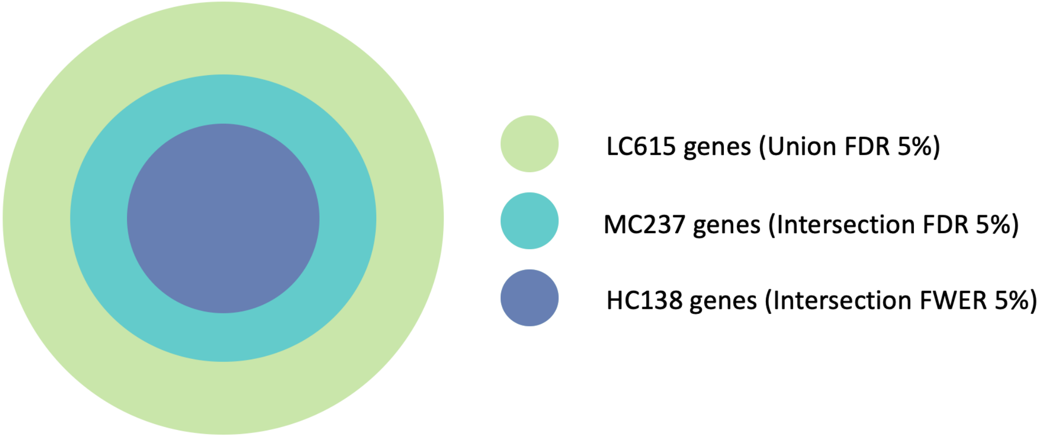


**Figure S3. Overlap of the three gene sets by different significant thresholds**. LC615 genes are the most comprehensive set of 615 genes with lowest confidence based on the union FDR 5% significance by one or more of three models, which includes the MC237 and HC138 genes; MC237 genes are 237 genes with moderate confidence based on the intersection FDR 5% significance by all three models—it is a subset of the LC615 genes, but includes all HC138 genes; HC138 genes are the most stringent of 138 genes with the highest confidence based on the intersection FWER 5% significance by all three models—it is a subset of the HC237 and LC615 genes.

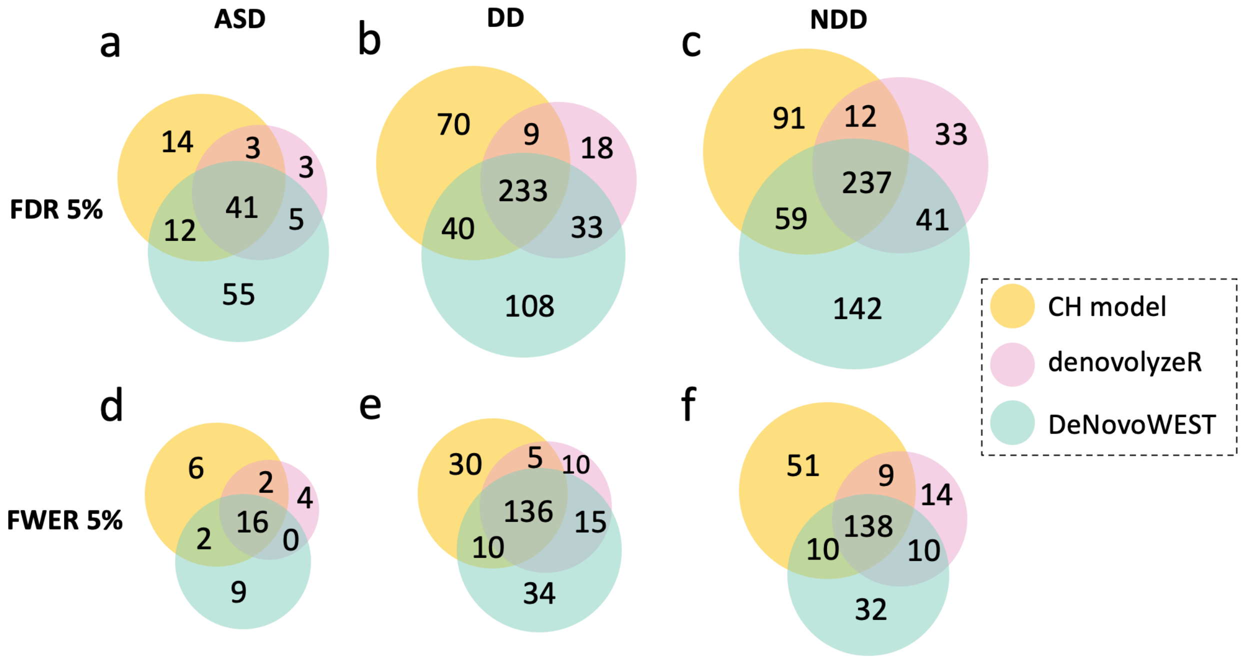


**Figure S4. Significant genes across phenotype by three models.** Overlap of FDR 5% significant genes by three models are shown in **(a)** ASD, **(b)** DD, and **(c)** NDD groups in the above panel; similarly, the overlap of FWER 5% significant genes are shown in **(d)** ASD, **(e)** DD, and **(f)** NDD groups.

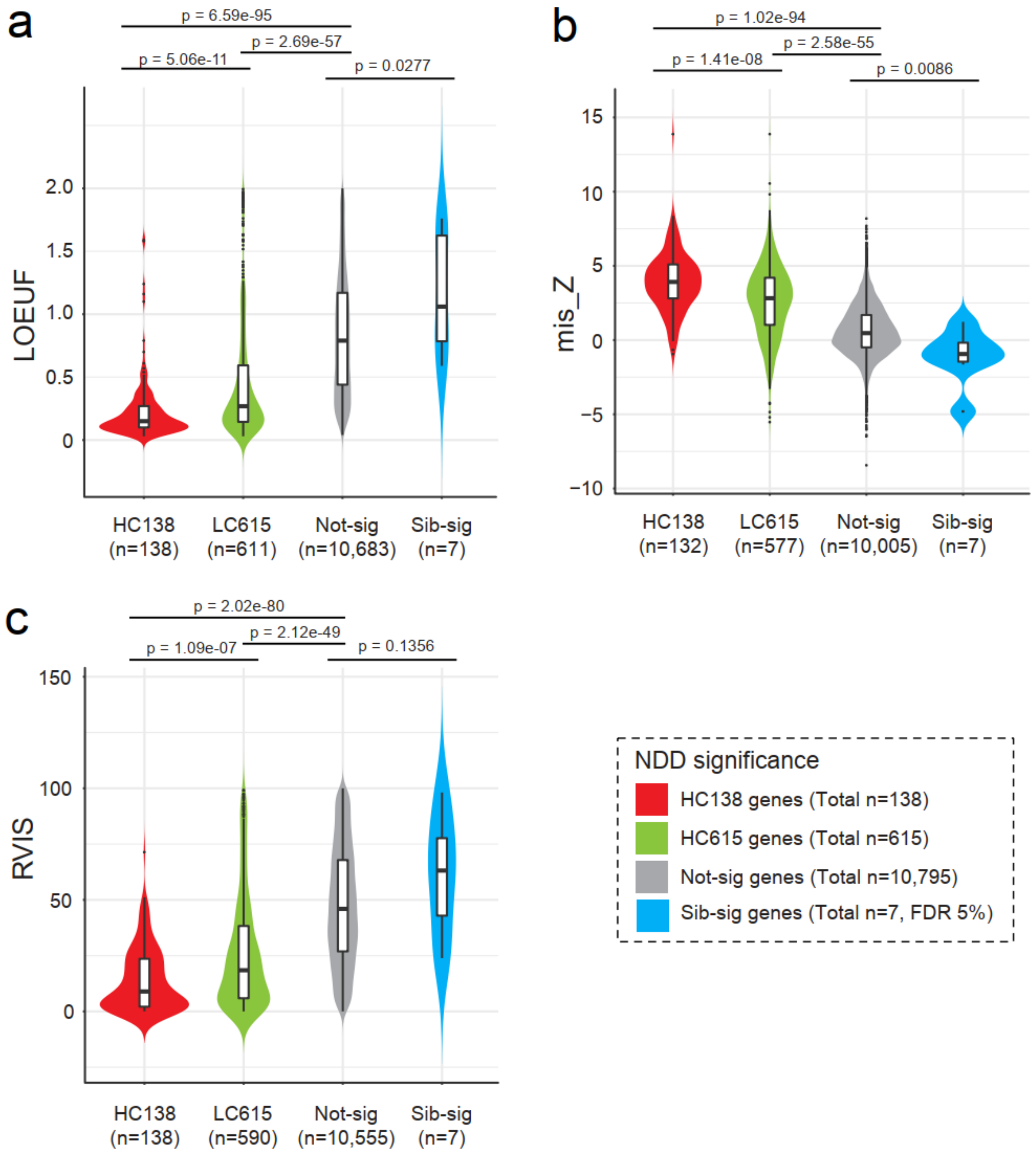


**Figure S5. Significant genes are highly enriched as intolerant genes.** Three intolerant score metrics—**(a)** LOEUF, **(b)** mis_Z, and **(c)** RVIS—were compared across HC138 and LC615 genes; the rest of genes with DNVs in the combined NDD group that did not show any DNV significance (Not-sig), and the genes show significance in siblings (Sib-sig). The LC615 and HC138 genes identified in this study are significantly intolerant to mutation when compared to not-significant genes, and also between the LC615 and HC138 significant genes, with the corresponding p-value annotated on the top. The number of genes with scores available are shown in parentheses below each category. Wilcoxon two-sample test was performed in R (v3.6.1) with the wilcox.test function. For the box plots, the lower whisker indicates the lowest data point excluding outliers (minima) and the upper whisker indicates the largest data point excluding outliers (maxima); the lower bound indicates the first quartile, which is the median of the lower half of the dataset (25th percentile), the upper bound indicates the third quartile, which is the median of the upper half of the dataset (75th percentile), and the middle value of the dataset (50th percentile) indicates in the middle.

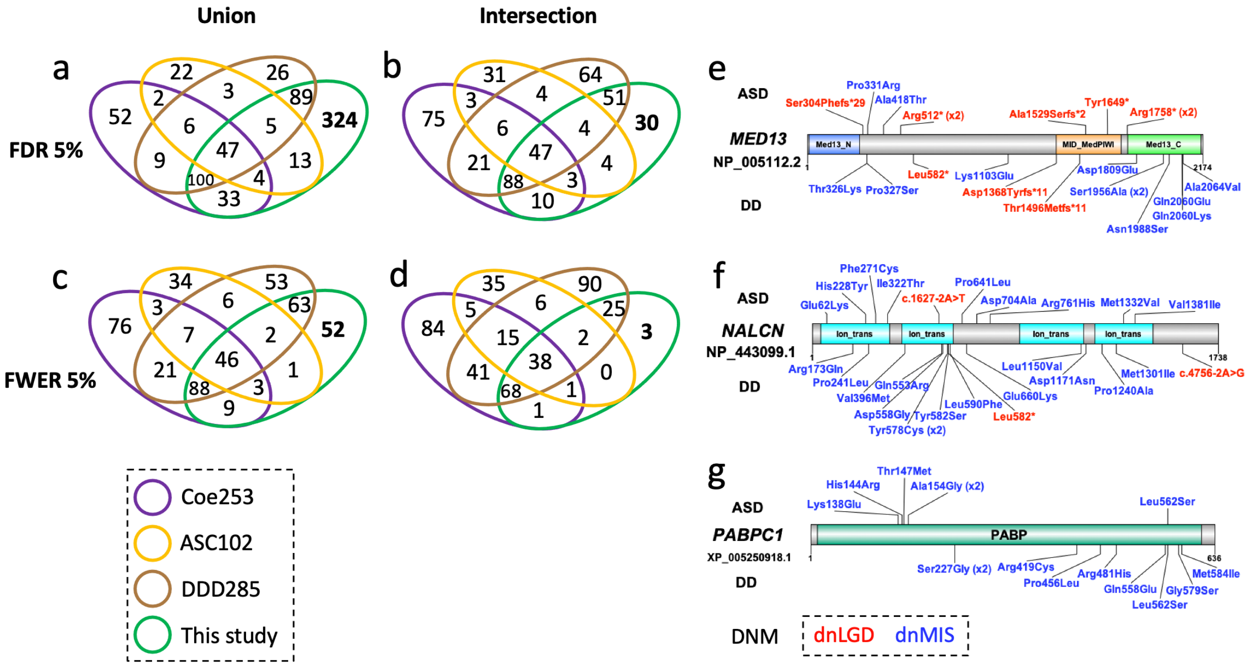


**Figure S6. Overlap of NDD-significant genes with reported significant genes.** Three main published gene lists—Coe253[20], ASC102[1], and DDD285[9]—were considered for the first-step comparison of reported significance. The overlap of these published genes with the genes identified in this study with FDR 5% significance at the **(a)** union set and **(b)** the intersection set of the three models (CH model, denovolyzeR, DeNovoWEST) are shown above; similarly, the overlap with FWER 5% significance in the **(c)** union set and **(d)** intersection set of all three models are shown below. If considered the most stringent of FWER 5% intersection significance, three candidate genes show potential "novel" statistical significance. Protein diagrams with dnLGD (red) and dnMIS (blue) variants from ASD (above the diagram, n = 15,560) and DD (below the diagram, n = 31,052) patients for the three candidates are shown in: **(e)** *MED13*, **(f)** *NALCN*, and **(g)** *PABPC1*.

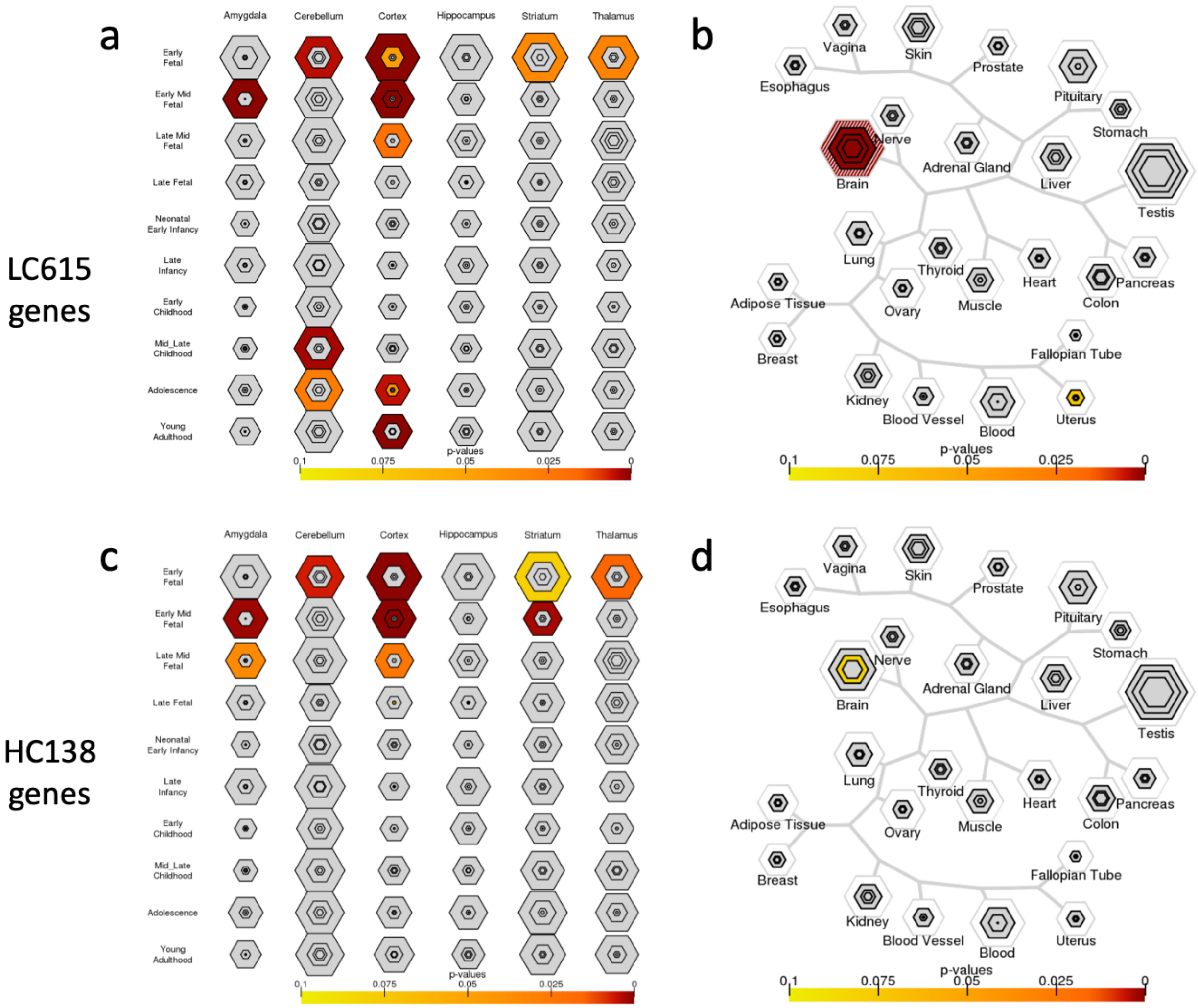


**Figure S7. Brain expression of significant genes. (a)** Specific brain region enrichment during development as determined by CSEA, showing enriched expression of the LC615 genes in the early-mid and late-mid fetal amygdala, early fetal cerebellum, early to late fetal cortex, early to early-mid fetal striatum, and early fetal thalamus. **(b)** Those genes have an enriched expression in the brain as suggested by TSEA. The p-values are from the Fisher's exact test followed by the Benjamini–Hochberg correction. Similar enrichment is also observed for the HC138 genes even with much fewer genes as shown in **(c)** and **(d)**.

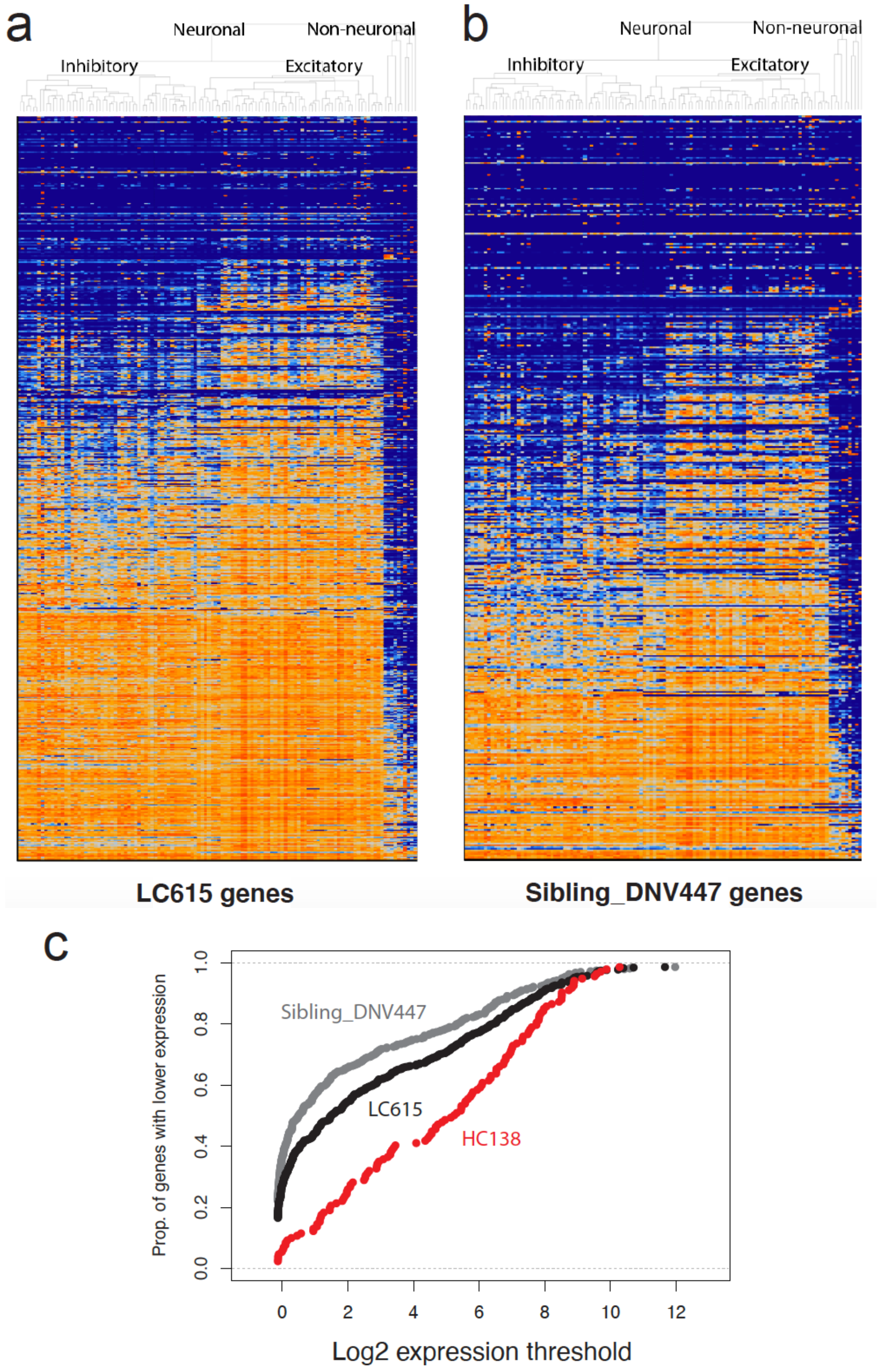


**Figure S8. Expression of NDD and control genes in human cortex.** Expression heatmap of **(a)** LC615 NDD genes and **(b)** 447 sibling DNV (n > 1) genes in 120 cell types identified across six human neocortical areas. **(c)** Empirical cumulative distributions of log2 trimmed mean expression of three gene sets. Rightward-shifted curves indicate more genes with greater expression.

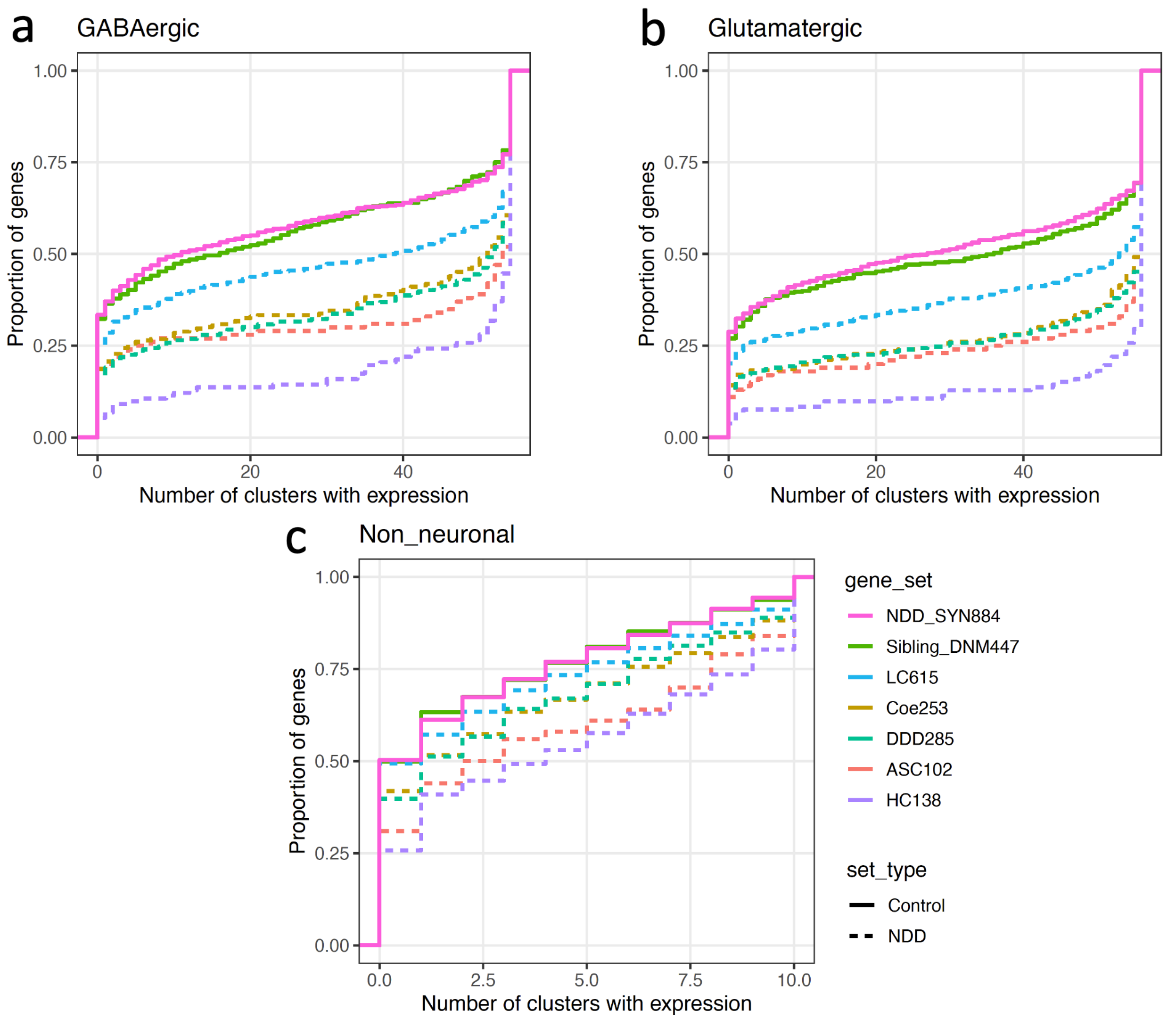


**Figure S9. Expression comparison of identified and reported NDD genes with control genes.** All NDD gene sets are significantly enriched in neuronal types compared to controls. The HC138 genes trend toward more neuronal enrichment than ASC102, although this is not statistically significant. All NDD gene sets (except the LC615 genes) are significantly enriched in non-neuronal cells compared to controls.

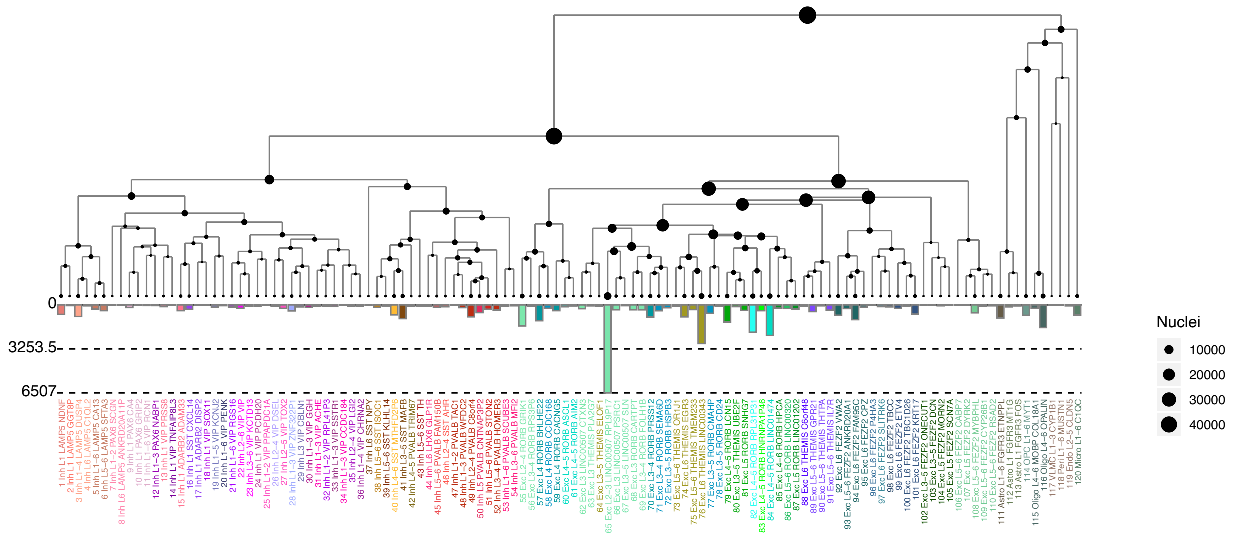


**Figure S10. The cell types and number of nuclei in single-nucleus RNA expression analysis.** The dendrogram below depicts the transcriptomic similarity between cell types (columns), the number of nuclei in each cell type (bar plot), and the cell-type labels that provide cortical layer and gene marker information.

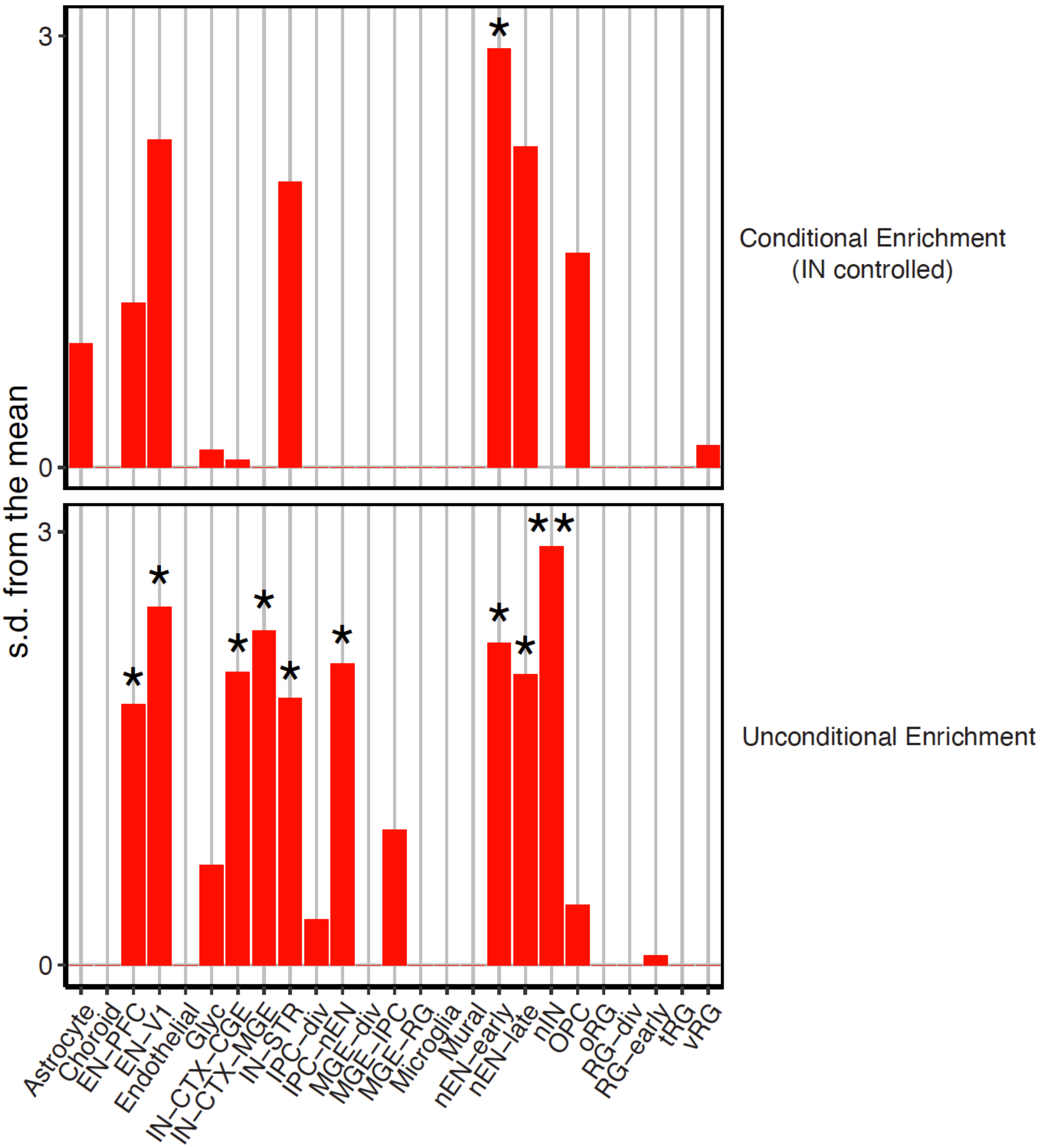


**Figure S11. Conditional and unconditional enrichment analyses.** Conditional enrichment analysis between the neuronal lineage populations found excitatory neurons to have an independent signal with respect to controlling interneuron signal; while neither enrichment nor significance ($p = 1$ for all) is reached in any neuronal lineage population when controlling for excitatory neurons (* indicates $p < 0.05$, ** indicates FDR $p < 0.05$).

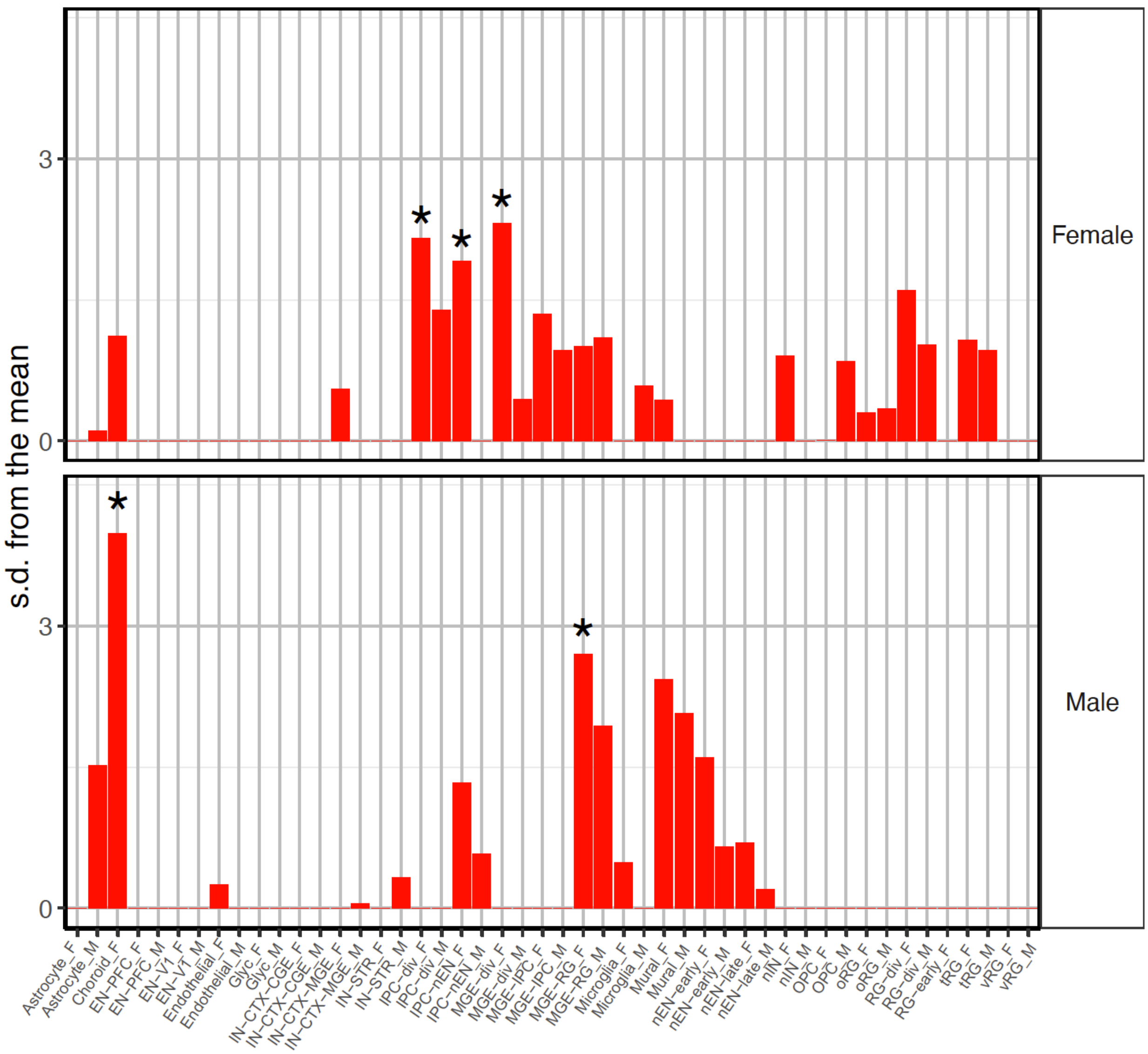


**Figure S12. Enriched expression of top genes with sex-biased DNV enrichment significance.** The top genes for both female and male DNV enrichment were tested with the HC138 genes as background. Enrichment signal was found in the intermediate progenitor cells for the female-enriched genes, and no such signal for male-enriched genes. Asterisk indicates $p < 0.05$; F means female and M indicates male in the legends on x-axis.

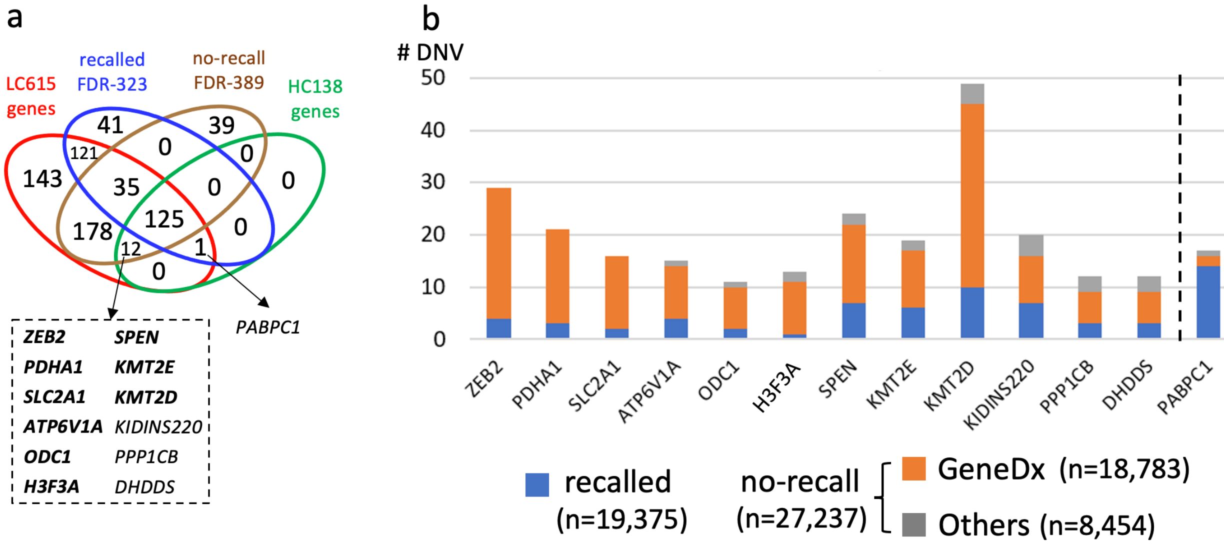


**Figure S13. Overlap of significant genes in NDD patients with recalled and no-recall subsets. (a)** Among the HC138 genes in the combined NDD group, there are 12 genes exclusively significant in the no-recall subset and one gene (*PABPC1*) exclusively significant in the recalled subset. **(b)** DNVs were almost exclusively from GeneDx, especially for *ZEB2*, *PDHA1*, and *SLC2A1* where all DNVs are from GeneDx and no DNVs from other cohorts in the no-recall subset. Note, the bar plot indicates the number of DNVs in each gene, they are absolute values and no statistics or distributions are involved.

### Supplementary Analyses

As ASD (n = 15,560) has a smaller sample size than the DD cohorts (n = 31,052), in order to model this effect for a more balanced sample size between ASD and DD cohorts, we have also performed two analyses by either downsampling DD samples or increasing ASD samples. In the analysis of downsampling DD samples, the data clearly show that the DD shows a greater degree of burden for DNV mutations, especially for dnLGD variants. We also performed another analysis with increasing ASD samples by adding the new release of SPARK_WES_2 and SPARK_WES_3. Both analyses with matched sample sizes for ASD and DD cohorts showed that sample size alone unlikely underlies the paucity of true ASD-specific genes at least for a *de novo* mutation model.

### I. Downsampling of DD to match ASD samples

First, we performed the downsampling of DD samples to match the number of ASD families. Specifically, we focused on the DDD13K and RUMC cohorts as we have the underlying sample manifest data for a total of 12,269 DD trios compared to 12,902 ASD trios from SSC, SPARK_WES_1, and ASC (Table S4).

| Phenotype | Cohort | Proband |
|---|---|---|
| **ASD** | SSC | 2,299 |
| | SPARK_WES_1 | 6,557 |
| | ASC | 4,046 |
| | **Subtotal** | **12,902** |
| | | |
| **DD** | DDD13K | 9,852 |
| | RUMC | 2,417 |
| | **Subtotal** | **12,269** |
| | | |
| **NDD** | **Total** | **25,171** |

**Table S4. Downsampled DD and size-matched ASD cohorts used in analysis.**

We repeated *de novo* enrichment analyses using the same three models (CH model, denovolyzeR, and DeNovoWEST) as we did for the entire DD, ASD, and the combined NDD cohorts. We considered the union and intersection of both the FDR 5% and FWER 5% significance, which are the same criteria used as in the manuscript. We found no ASD-specific

genes with strong significance, as the Venn shown below, even at a comparable sample size between ASD (n = 12,902) and DD (n = 12,269) cohorts. The number of DD genes was more than double that of ASD genes, including the discovery of DD-specific genes where no significant DNV enrichment was observed in ASD samples (Figure S14).

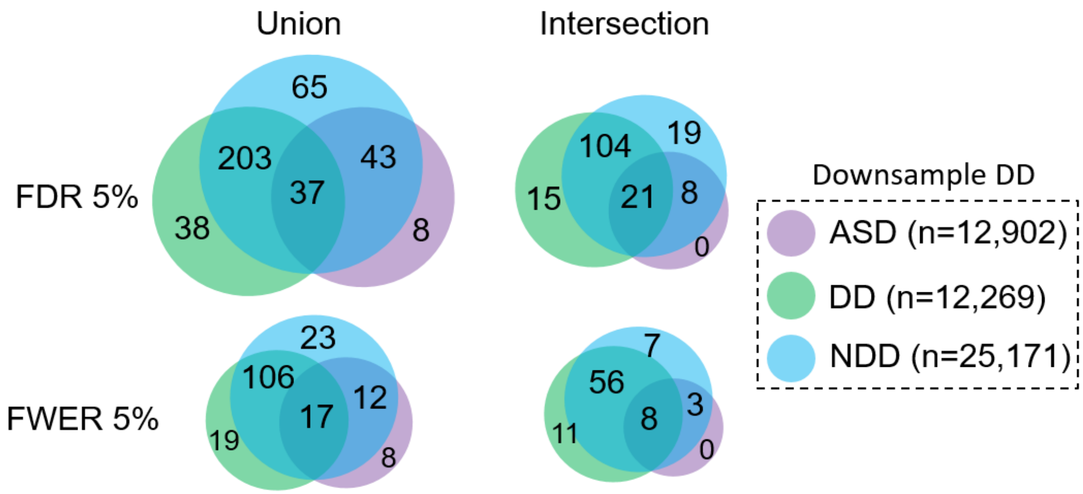


**Figure S14. Overlap of significant genes in the downsampled DD analysis.**

**II. Increase in the number of ASD families**

As a second approach, we also analyzed recently released ASD trio data from SPARK_WES_2 (n = 2,192) and SPARK_WES_3 (n = 2,060) as the reviewer suggested and used the same pipeline to identify DNVs as in SPARK_WES_1. We want to note that the majority of families in SPARK_WES_2 and SPARK_WES_3 are singletons that only have one or no parental DNA available to detect DNV. Taken together, the ASD size has increased to 19,812 trios by combining with all ASD cohorts in the present study (Table S5).

As for the DD cohorts, we have three cohorts included in the current study: DDD13K (n = 9,852), RUMC (n = 2,417), and GeneDx (n = 18,783). Due to the unavailability of the complete manifest for all samples (e.g., GeneDx), we are unable to perform a random downsampling of the DD samples. To match the ASD proband size, we took the combined ASD set (n = 19,812) and the GeneDx cohort (n = 18,783) to repeat the same *de novo* enrichment analyses.

| Phenotype | Cohort | Proband |
| --- | --- | --- |
| ASD | All ASD cohorts in present study | 15,560 |
| | SPARK_WES_2 | 2,192 |
| | SPARK_WES_3 | 2,060 |
| | **Subtotal** | **19,812** |
| | | |
| **DD** | **GeneDx** | **18,783** |
| | | |
| **NDD** | **Total** | **38,595** |

**Table S5. Increased ASD and size-matched DD cohorts used in analysis.** The SPARK_WES_2 and SPARK_WES_3 in red are new cohorts added here to increase the ASD size.

After applying the same thresholds of significance, we identified only one gene, *SMARCC2*, that met the most stringent threshold (FWER 5% intersection) specifically in ASD cohorts (Figure S15). *SMARCC2* loss-of-function and severe mutations, however, are known to be associated with Coffin-Siris syndrome-8 (OMIM #618362)—a known neurodevelopmental disorder. Indeed, we do observe two dnMIS variants among the DD cohort (n = 18,783) compared to seven dnLGD variants in the ASD cohorts (n = 19,912), so this does not represent a true ASD-specific gene. This is in contrast to several DD-specific genes already described in the main text.

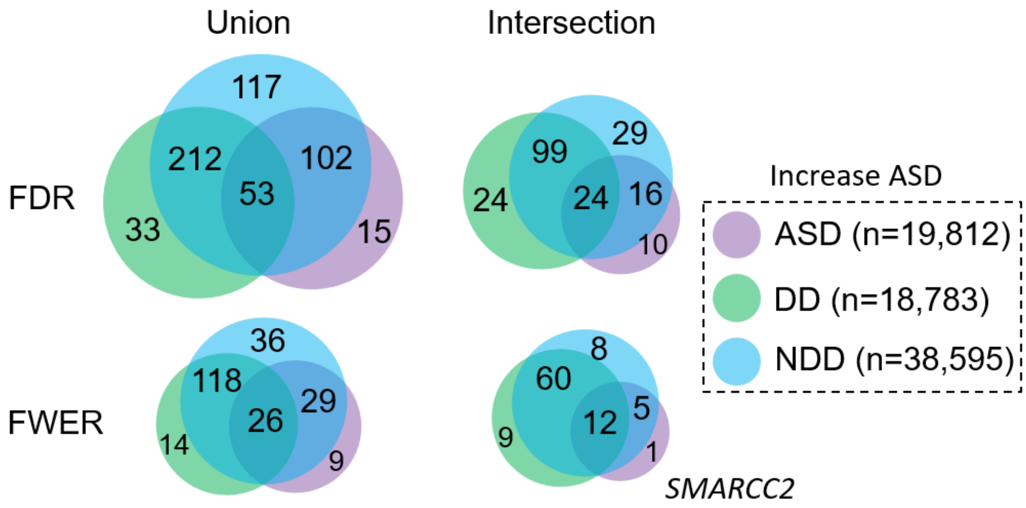


**Figure S15. Overlap of significant genes in enrichment analysis with increased ASD sample size.**

Thus, sample size unlikely underlies the paucity of true ASD-specific genes at least for a *de novo* mutation model.

## Supplementary References


1. Satterstrom, F.K. *et al.* Large-Scale Exome Sequencing Study Implicates Both Developmental and Functional Changes in the Neurobiology of Autism. *Cell* **180**, 568-584 e23 (2020).
2. Manichaikul, A. *et al.* Robust relationship inference in genome-wide association studies. *Bioinformatics* **26**, 2867-73 (2010).
3. McKenna, A. *et al.* The Genome Analysis Toolkit: a MapReduce framework for analyzing next-generation DNA sequencing data. *Genome Res* **20**, 1297-303 (2010).
4. Garrison, E. & Marth, G. Haplotype-based variant detection from short-read sequencing. (2012).
5. Rimmer, A. *et al.* Integrating mapping-, assembly- and haplotype-based approaches for calling variants in clinical sequencing applications. *Nat Genet* **46**, 912-918 (2014).
6. Kim, S. *et al.* Strelka2: fast and accurate calling of germline and somatic variants. *Nat Methods* **15**, 591-594 (2018).
7. Turner, T.N. *et al.* Sex-Based Analysis of De Novo Variants in Neurodevelopmental Disorders. *Am J Hum Genet* **105**, 1274-1285 (2019).
8. Bailey, J.A. *et al.* Recent segmental duplications in the human genome. *Science* **297**, 1003-7 (2002).
9. Kaplanis, J. *et al.* Evidence for 28 genetic disorders discovered by combining healthcare and research data. *Nature* (2020).
10. Chin, C.H. *et al.* cytoHubba: identifying hub objects and sub-networks from complex interactome. *BMC Syst Biol* **8 Suppl 4**, S11 (2014).
11. Xu, X., Wells, A.B., O'Brien, D.R., Nehorai, A. & Dougherty, J.D. Cell type-specific expression analysis to identify putative cellular mechanisms for neurogenetic disorders. *J Neurosci* **34**, 1420-31 (2014).
12. Zhou, X. *et al.* Integrating de novo and inherited variants in 42,607 autism cases identifies mutations in new moderate-risk genes. *Nature Genetics* (2022).
13. Fu, J.M. *et al.* Rare coding variation provides insight into the genetic architecture and phenotypic context of autism. *Nature Genetics* (2022).
14. Wilfert, A.B. *et al.* Recent ultra-rare inherited variants implicate new autism candidate risk genes. *Nat Genet* **53**, 1125-1134 (2021).
15. Guo, H. *et al.* Genome sequencing identifies multiple deleterious variants in autism patients with more severe phenotypes. *Genet Med* **21**, 1611-1620 (2019).
16. Feliciano, P. *et al.* Exome sequencing of 457 autism families recruited online provides evidence for autism risk genes. *NPJ Genom Med* **4**, 19 (2019).
17. RK, C.Y. *et al.* Whole genome sequencing resource identifies 18 new candidate genes for autism spectrum disorder. *Nat Neurosci* **20**, 602-611 (2017).
18. Takata, A. *et al.* Integrative Analyses of De Novo Mutations Provide Deeper Biological Insights into Autism Spectrum Disorder. *Cell Rep* **22**, 734-747 (2018).
19. Chen, R. *et al.* Leveraging blood serotonin as an endophenotype to identify de novo and rare variants involved in autism. *Mol Autism* **8**, 14 (2017).
20. Coe, B.P. *et al.* Neurodevelopmental disease genes implicated by de novo mutation and copy number variation morbidity. *Nature Genetics* **51**, 106-116 (2019).

PNAS

## The SPARK Consortium Authors

The following authors were part of the SPARK Consortium:

John Acampado [1], Andrea J. Ace [1], Alpha Amatya [1], Irina Astrovskaya [1], Asif Bashar [1], Elizabeth Brooks [1], Martin E. Butler [1], Lindsey A. Cartner [1], Wubin Chin [1], Wendy K. Chung [1,2], Amy M. Daniels [1], Pamela Feliciano [1], Chris Fleisch [1], Swami Ganesan [1], William Jensen [1], Alex E. Lash [1], Richard Marini [1], Vincent J. Myers [1], Eirene O'Connor [1], Chris Rigby [1], Beverly E. Robertson [1], Neelay Shah [1], Swapnil Shah [1], Emily Singer [1], LeeAnne G. Snyder [1], Alexandra N. Stephens [1], Jennifer Tjernagel [1], Brianna M. Vernoia [1], Natalia Volfovsky [1], Loran Casey White [1], Alexander Hsieh [2], Yufeng Shen [2], Xueya Zhou [2], Tychele N. Turner [3], Ethan Bahl [4], Taylor R. Thomas [4], Leo Brueggeman [4], Tanner Koomar [4], Jacob J. Michaelson [4], Brian J. O'Roak [5], Rebecca A. Barnard [5], Richard A. Gibbs [6], Donna Muzny [6], Aniko Sabo [6], Kelli L. Baalman Ahmed [6], Evan E. Eichler [7], Matthew Siegel [8], Leonard Abbeduto [9], David G. Amaral [9], Brittani A. Hilscher [9], Deana Li [9], Kaitlin Smith [9], Samantha Thompson [9], Charles Albright [10], Eric M. Butter [10], Sara Eldred [10], Nathan Hanna [10], Mark Jones [10], Daniel Lee Coury [10], Jessica Scherr [10], Taylor Pifher [10], Erin Roby [10], Brandy Dennis [10], Lorrin Higgins [10], Melissa Brown [10], Michael Alessandri [11], Anibal Gutierrez [11], Melissa N. Hale [11], Lynette M. Herbert [11], Hoa Lam Schneider [11], Giancarla David [11], Robert D. Annett [12], Dustin E. Sarver [12], Ivette Arriaga [13], Alexies Camba [13], Amanda C. Gulsrud [13], Monica Haley [13], James T. McCracken [13], Sophia Sandhu [13], Maira Tafolla [13], Wha S. Yang [13], Laura A. Carpenter [14], Catherine C. Bradley [14], Frampton Gwynette [14], Patricia Manning [15], Rebecca Shaffer [15], Carrie Thomas [15], Raphael A. Bernier [16], Emily A. Fox [16], Jennifer A. Gerdts [16], Micah Pepper [16], Theodore Ho [16], Daniel Cho [16], Joseph Piven [17], Holly Lechniak [18], Latha V. Soorya [18], Rachel Gordon [18], Allison Wainer [18], Lisa Yeh [18], Cesar Ochoa-Lubinoff [19], Nicole Russo [19], Elizabeth Berry-Kravis [20], Stephanie Booker [21], Craig A. Erickson [21], Lisa M. Prock [22], Katherine G. Pawlowski [22], Emily T. Matthews [22], Stephanie J. Brewster [22], Margaret A. Hojlo [22], Evi Abada [22], Elena Lamarche [23], Tianyun Wang [24], Shwetha C. Murali [7], William T. Harvey [24], Hannah E. Kaplan [25], Karen L. Pierce [25], Lindsey DeMarco [26], Susannah Horner [26], Juhi Pandey [26], Samantha Plate [26], Mustafa Sahin [27], Katherine D. Riley [27], Erin Carmody [27], Julia Constantini [7], Amy Esler [28], Ali Fatemi [29], Hanna Hutter [29], Rebecca J. Landa [29], Alexander P. McKenzie [29], Jason Neely [29], Vini Singh [29], Bonnie Van Metre [29], Ericka L. Wodka [29], Eric J. Fombonne [30], Lark Y. Huang-Storms [30], Lillian D. Pacheco [30], Sarah A. Mastel [30], Leigh A. Coppola [30], Sunday Francis [31], Andrea Jarrett [31], Suma Jacob [31], Natasha Lillie [31], Jaclyn Gunderson [31], Dalia Istephanous [31], Laura Simon [31], Ori Wasserberg [31], Angela L. Rachubinski [32], Cordelia R. Rosenberg [32], Stephen M. Kanne [33,34], Amanda D. Shocklee [34], Nicole Takahashi [34], Shelby L. Bridwell [34], Rebecca L. Klimczac [34], Melissa A. Mahurin [34], Hannah E. Cotrell [34], Cortaiga A. Grant [34], Samantha G. Hunter [34], Christa Lese Martin [35], Cora M. Taylor [35], Lauren K. Walsh [35], Katherine A. Dent [35], Andrew Mason [36], Anthony Sziklay [36], Christopher J. Smith [36]

[1] Simons Foundation, New York, USA
[2] Columbia University, New York, USA
[3] Washington University School of Medicine, St. Louis, USA
[4] University of Iowa Carver College of Medicine, Iowa City, USA
[5] Oregon Health & Science University, Portland, USA
[6] Baylor College of Medicine, Houston, USA
[7] University of Washington School of Medicine & Howard Hughes Medical Institute, Seattle, USA
[8] Maine Medical Center Research Institute, Portland, USA
[9] University of California, Davis, Sacramento, USA
[10] Nationwide Children's Hospital, Columbus, USA
[11] University of Miami, Coral Gables, USA
[12] University of Mississippi Medical Center, Jackson, USA
[13] University of California, Los Angeles, Los Angeles, USA
[14] Medical University of Southern Carolina (MUSC), Portland, USA

[15] Cincinnati Children's Hospital Medical Center - Research Foundation, Cincinnati, USA
[16] Seattle Children's Autism Center/UW, Seattle, USA
[17] University of North Carolina at Chapel Hill, Chapel Hill, USA
[18] Department of Child & Adolescent Psychiatry, Rush University Medical Center, Chicago, USA
[19] Department of Developmental & Behavioral Pediatrics, Rush University Medical Center, Chicago, USA
[20] Department of Neurological Sciences, Department of Pediatrics, Department of Biochemistry, Rush University Medical Center, Chicago, USA
[21] Cincinnati Children's Hospital Medical Center - Research Foundation, Cincinnati, USA
[22] Boston Children's Hospital (BCH), Boston, USA
[23] University of North Carolina at Chapel Hill, Chapel Hill, USA
[24] University of Washington School of Medicine, Seattle, USA
[25] University of California, San Diego, School of Medicine, La Jolla, USA
[26] Children's Hospital of Philadelphia, Philadelphia, USA
[27] Boston Children's Hospital (BCH), Boston, USA
[28] University of Minnesota, Minneapolis, USA
[29] Kennedy Krieger Institute, Baltimore, USA
[30] Oregon Health & Science University, Portland, USA
[31] University of Minnesota, Minneapolis, USA
[32] University of Colorado School of Medicine, Aurora, USA
[33] Department of Health Psychology, University of Missouri, Columbia, USA
[34] Thompson Center for Autism and Neurodevelopmental Disorders, University of Missouri, Columbia, USA
[35] Geisinger Autism & Developmental Medicine Institute, Lewisburg, USA
[36] Southwest Autism Research and Resource Center, Phoenix, USA

The SPARK Consortium authors coordinated the samples and sequencing for the SPARK cohort.
